# The whole genomic analysis of the Orf virus strains ORFV-SC and ORFV-SC1 from the Sichuan province and their weak pathological response in rabbits

**DOI:** 10.1007/s10142-023-01079-z

**Published:** 2023-05-16

**Authors:** Guoyu Du, Jinyan Wu, Cheng Zhang, Xiaoan Cao, Lingxia Li, Jijun He, Yong Zhang, Youjun Shang

**Affiliations:** 1 College of Veterinary Medicine, Gansu Agricultural University, Lanzhou, 730046 China; 2 State Key Laboratory for Animal Disease Control and Prevention, Lanzhou Veterinary Research Institute, Chinese Academy of Agricultural Sciences, Lanzhou, 730046 China; 3 State Key Laboratory of Veterinary Etiological Biology, Lanzhou Institute of Veterinary Research (CAAS) Institute, Chinese Academy of Agricultural Sciences, Lanzhou, 730046 China

**Keywords:** Orf virus, Genome sequencing, Phylogenetic tree, Secondary and tertiary structure, ORFV-attenuated vaccines

## Abstract

The Orf virus (ORFV) is a member of the Parapoxvirus genus of the Poxviridae family and can cause contagious diseases in sheep, goats, and wild ungulates. In the present study, two ORFV isolates (ORFV-SC isolated from Sichuan province and ORFV-SC1 produced by 60 passages of ORFV-SC in cells) were sequenced and compared to multiple ORFVs. The two ORFV sequences had entire genome sizes of 14,0707 bp and 141,154 bp, respectively, containing 130 and 131 genes, with a G + C content of 63% for the ORFV-SC sequence and 63.9% for the ORFV-SC1 sequence. Alignment of ORFV-SC and ORFV-SC1 with five other ORFV isolates revealed that ORFV-SC, ORFV-SC1, and NA1/11 shared > 95% nucleotide identity with 109 genes. Five genes (ORF007, ORF20, ORF080, ORF112, ORF116) have low amino acids identity between ORFV-SC and ORFV-SC1. Mutations in amino acids result in changes in the secondary and tertiary structure of ORF007, ORF020, and ORF112 proteins. The phylogenetic tree based on the complete genome sequence and 37 single genes revealed that the two ORFV isolates originated from sheep. Finally, animal experiments demonstrated that ORFV-SC1 is less harmful to rabbits than ORFV-SC. The exploration of two full-length viral genome sequences provides valuable information in ORFV biology and epidemiology research. Furthermore, ORFV-SC1 demonstrated an acceptable safety profile following animal vaccination, indicating its potential as a live ORFV vaccine.

**Supplementary Information:**

The online version contains supplementary material available at 10.1007/s10142-023-01079-z.

## Background

Sheep and goats are affected by Orf, a non-systemic skin condition caused by an acute viral skin infection. Erythema, papules, infectious abscesses, ulcers, scabs on the lips, oral and nasal mucosa, skin, and udders are the most common symptoms in lambs. Orf is a zoonosis with a global distribution that affects humans, especially animal workers such as farmers and veterinarians, and other people in direct or indirect contact with sick animals (Andreani et al. 2019; Leavell et al. 1968; Lederman et al. 2007). The disease not only has a negative economic impact on the breeding industry but also puts animal health at risk. The Orf death rate in sheep and goats is usually low but might increase if complicated by bacterial and fungal infections (Bala et al. 2018). Furthermore, the lesions on the lips and oral cavity hinder the feeding of animals. Orf virus is a large, enclosed, double-stranded DNA virus and is a member of the parapoxvirus genus, which belongs to the Poxviridae subfamily chordopoxvirinae. This family also includes the cattle pseudocowpox virus (PCPV), bovine papular stomatitis virus (BPSV), squirrel parapoxvirus (SPPV), and red deer parapoxvirus (PVNZ) (Hosamani et al. 2006; Zhang et al. 2010). Compared to other poxviruses, the Orf virus has a small genome of about 139 kbp long, with a G + C content of 64% and 130 putative genes, including 89 central conserved genes and terminal variable genes (Chen et al. 2017; Mercer et al. 2006). Numerous whole or almost complete ORFV genomic sequences have been released in the GenBank database. However, the genome sequences of ORFV vaccine strains are rarely accessible in GenBanks, with only one describing the sequencing of the live Ecthyma vaccine (Heare et al. 2020). The vaccination strains may vary in sequence over time, and a change in one of the virulence genes may result in increased vaccine virulence.

In China, Orf outbreaks were recently reported in Jilin, Fujian, Shandong, Anhui, etc. Although a live ecthyma vaccine is used in some regions in China, genetic data regarding this vaccine is scarce (Schmidt et al. 2013). This study aimed to present the detection and complete sequence of the ORFV strain (named ORFV-SC) successfully isolated from Sichuan, China. The ORFV-SC strain was attenuated by performing 60 continuous passages in cells, yielding another strain (ORFV-SC1). In addition, data related to the genome structure and phylogenetic relationship, examination of genomic variation between the two ORFV and other ORFV isolates published in GenBank, and further verification of the safety of ORFV-SC1 as a vaccine are discussed. The findings provide a deeper understanding of the diversity of ORFV isolates circulating in this region and around the world. The ORFV-SC1 genome-wide information elaborates on the genetic variation of attenuated ORFV and wild isolates and contributes to the study of ORFV live vaccines.

## Materials and methods

### Tissue sample

In March 2017, a natural ORFV outbreak occurred in a herd of 133 sheep in Nanchong of Sichuan Province, southwest China. The morbidity rate of this outbreak was 12.7% (17 out of 133). This ORFV strain infected lambs aged 1 to 4 months, and a small number of adult sheep presented with typical Orf clinical features, including papules, pustules, and scabs on the lips. The clinical lip scab samples were collected from lambs suspected to be infected with ORFV and were stored at − 80 ℃.

### Virus isolation and immunofluorescence assay

The collected scab tissue samples were triturated in 0.01 M PBS over two hours at 4 ℃. The samples were centrifuged at 4000 r/min for 10 min at 4 ℃ and then further centrifuged at 12,000 r/min for 10 min at 4 ℃; 100 μl of the supernatant was filtered with a 0.22-μm filter, and 3 ml of maintenance medium was added to sheep fetal fibroblasts. The inoculated cells were then placed in a CO_2_ incubator with 5% CO_2_, and 7 ml of maintenance medium was added after 2 h. Normal cells were maintained similarly to the control. Noticeable cytopathic effects (CPE) were observed in the first generation of cells inoculated with the virus. Cells with significant CPE were collected and frozen and thawed twice, and we used the Reed-Muench method to calculate the TCID_50_ of ORFV-SC passage 5. Sheep fetal fibroblasts were infected with a 0.1 MOI dose of the virus in T25 cell flasks for serial passage (60 passages). The time of all lesions after the cells were inoculated with the virus was observed and recorded, and the TCID_50_ of the 60th passage virus was detected, as described above.

In addition, the CPE-positive cells were fixed with 4% paraformaldehyde after washing three times with PBS (PH = 7.2). The cells were then incubated with 5% skimmed milk powder at 37 ℃ for 1 h. Goat ORFV-positive serum, camel goatpoxvirus (GTPV)-positive serum, goat foot–and–mouth disease (FMDV)-positive serum, and bovine lumpy skin disease virus (LSDV)-positive stored in our laboratory were added as the primary antibodies, and rabbit anti-goat FITC antibodies, goat anti-camel FITC antibodies, and rabbit anti-bovine FITC antibodies were used as the secondary antibodies. Cells without the Orf virus were used as the control group. The primary antibody was diluted at 1:500 and incubated at 37 ℃ for 2 h, while the secondary antibody was diluted at 1:2000 and incubated at 37 ℃ for 1 h. The nuclei were stained with DAPI at 37 ℃ for 5 min and immediately observed and photographed using an inverted fluorescence microscope.

### DNA sequencing and assembly

The collected virions were purified by sucrose gradient ultracentrifugation, as described previously (Li et al. 2012). Viral DNA was extracted using the QIAGEN DNeasy Blood and Tissue Kit according to the manufacturer’s instructions. The virus was isolated from Sichuan sheep samples, which were passaged in cells for 60 passages, ORFV-SC passage 5 and passage 60 (ORFV-SC1) using HisSeq XTen for whole genome sequencing (Shanghai China). First, concentrations of ORFV-SC and ORFV-SC1 were determined using the Qubit assay and a high-sensitivity kit (Life Technologies, https://www.thermofisher.com) at 232 ng/ul and 132 ng/µL, respectively. For the preparation of paired-end libraries, high quantities of genomes were diluted to yield 1 ug of each genome as input. The DNA was broken up and tagged during the tagmentation process (Illumina). The tag adapters were finished by limited cycle PCR amplification (12 cycles), which also introduced dual-index barcodes. AMPure XP beads (Beckman Coulter Inc. https://www.beckmancoulter.com), libraries on specific beads, were normalized according to the Nextera XT protocol (Illumina). For sequencing, the standardized libraries were combined into a single library. The combined single-strand library was then placed into the kit, loaded onto the instrument, and loaded onto the flow cell. Bi-exponential reads were used for automated cluster creation and paired-end sequencing, completing 2 × 250 bp in a single 39-h run. Raw data were obtained by sequencing and then cleaned by removing artificial sequences using the fastp software. After removing the low-quality regions, contings were assembled, and scaffolds were constructed using clean data with MITObim (https://github.com/chrishah/MITObim). The complete genome sequences of ORFV-SC and ORFV-SC1 have been submitted to GenBank with accession numbers ON932451.1 and ON932452.1, respectively.

### Gene prediction and analysis

The gene sequences were predicted using the Genome Annotation Transfer Utility (GATU) software, and the prediction results were then compared. A gene was considered true if the positive-strand comparison stop codon and the negative-strand comparison start codon were the same and the identity percent was > 60. The true genes were retained (Frishman 2007). A blastp analysis (https://blast.ncbi.nlm.nih.gov/Blast.cgi) of all predicted proteins was performed against the nonredundant database at the National Center for Biotechnology Information (https://www.ncbi.nlm.nih.gov), and the gene annotation was referenced by downloading other known ORFV genomes from the NCBI (ftp://ftp.ncbi.nlm.nih.gov/genomes/archive/old_refseq/Bacteria/) GenBank (https://www.ncbi.nlm.nih.gov/genbank/) (listed in Table 1).

**Table 1:** 22 fully sequenced ORFVs were used in this study

Species	Isolate	Host	Country	No. of predicted genes	Genome size (bp)	GenBank accession no	Reference
ORFV	SC	Sheep	China	130	140,646	ON932451.1	Present study
ORFV	SC1	Sheep	China	131	140,952	ON932452.1	Present study
ORFV	NA1/11	Sheep	China	132	137,080	KF234407.1	
ORFV	GO	Goat	China	132	139,866	KP010354.1	Chi et al. 2015)
ORFV	SJ1	Goat	China	129	139,112	KP010356.1	Chi et al. 2015)
ORFV	OV-HN3/12	Sheep	China	132	136,643	KY053526	Zhang et al. 2010)
ORFV	SA00	Goat	USA	130	139,962	AY386264	Hua et al. 2015)
ORFV	NZ2	Sheep	New Zealand	132	137,820	DQ184476.1	Chen et al. 2017)
ORFV	IA82	Sheep	USA	130	137,241	AY386263.1	Hua et al. 2015)
ORFV	NA17	Goat	China	132	139,287	MG674916.2	Friederichs et al. 2014)
ORFV	SY17	Sheep	China	131	140,413	MG712417	Friederichs et al. 2014)
ORFV	MP	Goat	India	134	139,807	MT332357.1	
ORFV	CL18	Sheep	China	134	138,495	MN648219.1	
ORFV	TVL	Ovine	USA	134	134,893	MN454854.1	Li et al. 2012)
ORFV	NP	Goat	China	134	132,111	KP010355.1	
ORFV	YX	Goat	China	134	138,231	KP010353.1	
ORFV	B029	Homo	Germany	132	134,104	KF837136.1	
ORFV	UPM/HSN-20	Goat	Malaysia	131	132,124	MW537048.1	
PCPV	VR634	Reindeer	Finland	134	145,289	GQ329670.1	
PCPV	F00.120R	Reindeer	Finland	134	133,169	GQ329669.1	
BPSV	TX09c5	Bovine	USA	133	135,635	KM875471.1	
BPSV	AR02	Bovine	USA	134	134,431	AY386265.1	

### Phylogenetic analysis

The genetic evolution information of the ORFV-SC and ORFV-SC1 strains was obtained after sequencing. The two complete genome sequences were compared to those of ORFV isolates available in the GenBank database using the online BLAST program. Individual nucleotide sequences and complete genome sequences (22 PPV strains in total, listed in Table 1) were aligned by using Clustal W. Single gene sequences of TVL were not aligned because gene numbering was inconsistent with other PPVs. Subsequently, IQ-TREE software was used to construct phylogenetic trees based on the complete genome and individual genes. In this process, the neighbor-joining method was applied with 1000 bootstraps (Gelaye et al. 2016).

### Genome-wide comparison of ORFV-SC and ORFV-SC1 and prediction of protein structure

To confirm whether some genes of ORFV-SC were mutated in serial passages affecting viral virulence, the amino acid sequence of each ORF was analyzed using the software DNAstar in order to determine the mutations and deletions/insertions located in the sequences of ORFV-SC and ORFV-SC1. For genes with mutations in SC and SC1, SMART (Letunic and Bork 2018) (simple modular architecture research tool) (https://smart.embl.de/) was used to analyze their protein structure functional domains; protein secondary structure was predicted by SOPMA (self-optimized predicted method with alignment) (https://npsa-prabi.ibcp.fr/cgi-bin/npsa_automat.pl?page=npsa_sopma.html) (Gao et al. 2021); and finally, the protein tertiary structure constructed and analysis was performed using I-TASSER (Zhang et al. 2019) (iterative threading assembly refinement) (https://zhanggroup.org/I-TASSER/), and the models were visualized in pyMOL 2.5.4 (https://pymol.org/2/).

### Virological, clinicopathological monitoring, and pathological section

Fifteen rabbits aged 6–8 weeks were randomly divided into three groups, with 3 in the control group and 6 in each of the ORFV-SC and ORFV-SC1 groups. Group 1 (ORFV-SC strain, 10^3.25^ TCID50/ml) and group 2 (ORFV-SC1 strain, 10^5.5^ TCID50/ml) received 100 μl of the virus by oral site inoculation, while group 3 was injected with 100 ul of 1 × phosphate buffer solution (PBS) as a negative control. Tissues (lip, palate, heart, liver, spleen, kidney, lymph nodes) were collected from group 1 and group 2 rabbits on day 7 after injection. The samples were fixed in 4% paraformaldehyde, dehydrated, cleared, waxed, embedded in paraffin, cut into 4-μm-thick sections, and examined as previously described (Schmitz et al. 2010). Also, the tissues (lip, palate, heart, liver, spleen, kidney, lymph nodes) collected from group 1 and group 2 rabbits (3 in each group) were tested by the ORFV real-time PCR kit purchased from Beijing TIANDZ to compare viral loads in different organs of rabbits infected with wild isolate ORFV-SC and cell-passaged strain ORFV-SC1 (three rabbit tissues were collected from each experimental group and one from the control group). Two grams of each tissue sample were triturated in 0.01 M PBS overnight at 4 ℃ and centrifuged at 10,000 r/min for 10 min; 200 μl of the supernatant was used for DNA extraction. Then, the viral DNA samples were analyzed using the probe method real-time RT-qPCR; 25 μl of the PCR mixture contained 12.5 μl of premix ExTaqTM, 0.5 μl of each primer (10 μmol/L), 0.5 μl probe (10 μmol/L), 3 μl of template (15 ng/μl), and 8 μl of ddH2O. Real-time PCR was performed at 94 ℃ for 2 min, followed by 40 cycles at 94 ℃ for 20 s; annealing was carried out at 58 ℃ for 30 s and extension at 72 ℃ for 20 s. The cycle threshold (Ct) values of all samples were converted into copy numbers according to the standard curve *y* =  − 0.3259X + 13.755. Drawings were done using GraphPad Prism V5.0 software, and the data were analyzed by SPSS software.

For the remaining animals, monitoring was performed daily until week 4. Clinical monitoring was performed by detailed and standard examinations of the injection site. Any apparent visual changes at the injection site and adjacent sites were recorded on a single form, and body temperature, mental status, and feed intake were measured daily.

## Results

### Typical clinical characteristics

**Fig. 1:**
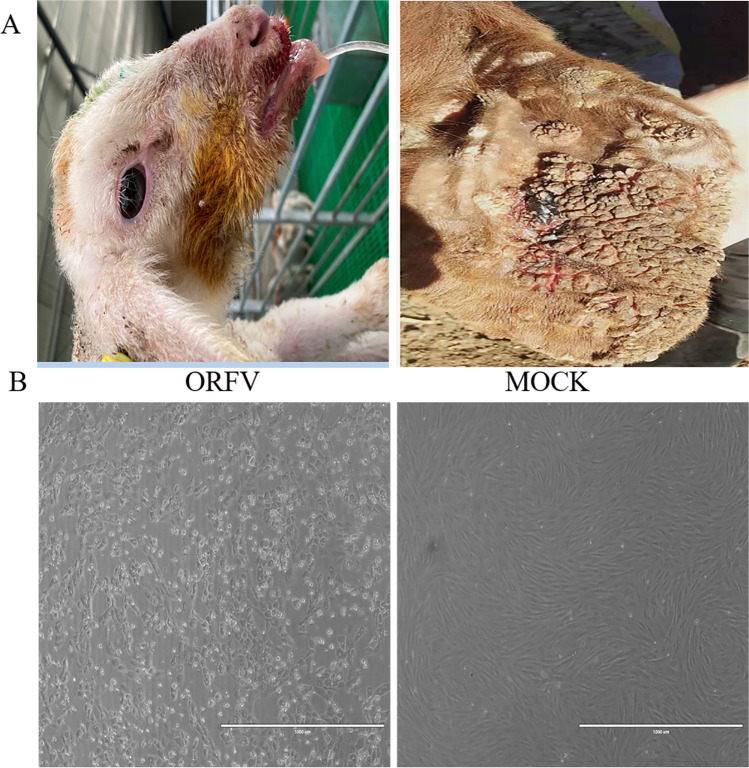
ORFV-induced lesions in animals and cells. (**A**) The picture on the left shows the appearance of ulcers in the snouts of lambs caused by ORFV, and the lambs are thin. On the right is a large number of cauliflower-shaped scabs on the ears of adult sheep caused by ORFV. (**B**) White light diagram of normal sheep fetal fibroblasts and lesion after affected ORFV

In March 2017, a suspected ORFV infection was reported on a farm in Nanchong city, Sichuan province; 17 sheep were infected in this suspected ORFV outbreak. The typical Orf clinical features were observed on the lips, muzzle, and nostrils (Fig. 1A). All infected sheep recovered in about a month, and no death was recorded. ORFV infection was initially diagnosed based on the above.

### Virus identification by immunofluorescence assay

**Fig. 2:**
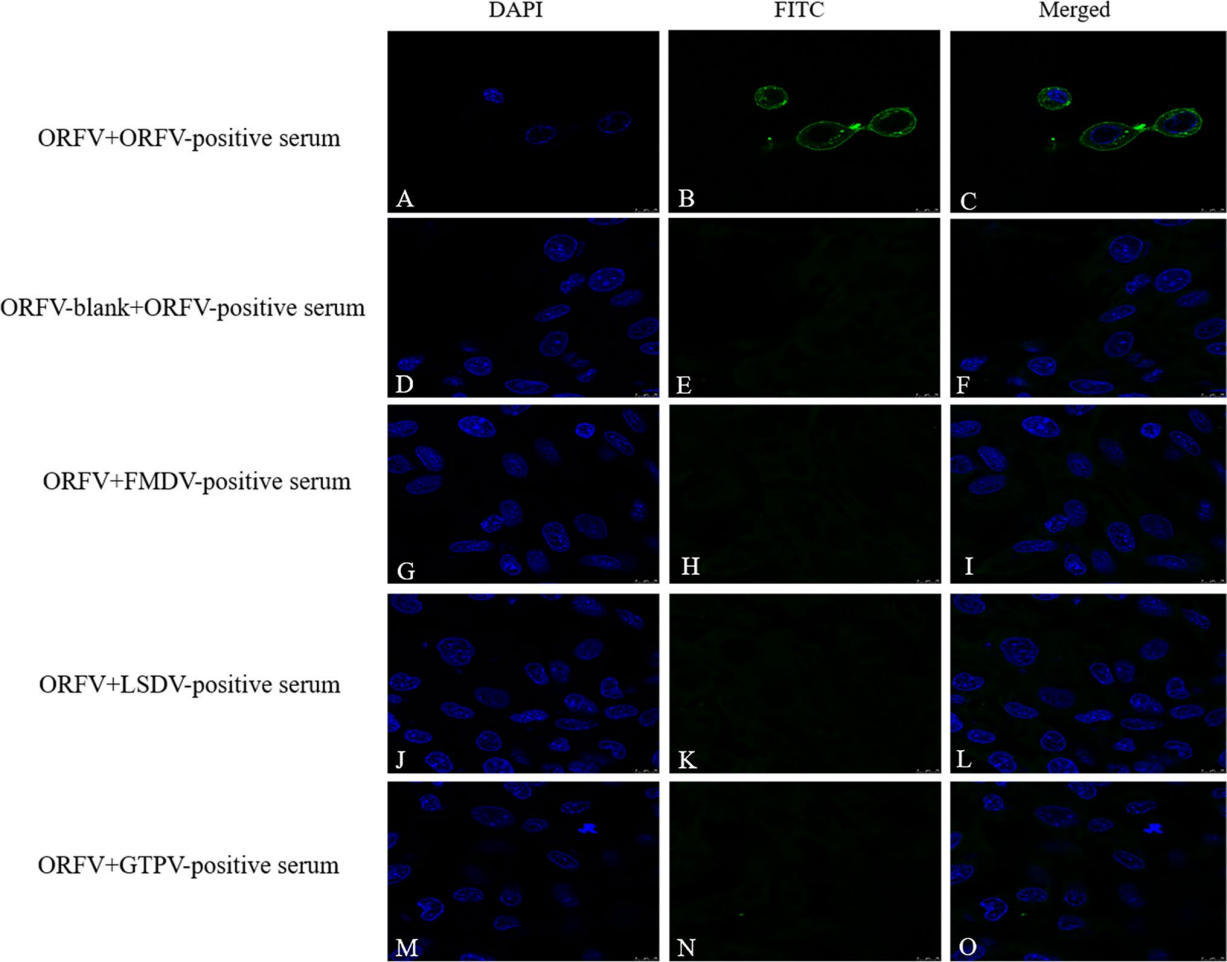
Identification of ORFV by indirect immunofluorescence assay. Sheep fetal fibroblasts were infected with ORFV at 37 °C for 36 h. As primary antibodies, ORFV, GTPV, LSDV, and FMDV-positive serum were used. Anti-goat IgG, anti-camel lgG, anti-bovine FITC as secondary antibody and DAPI was used to stain nuclei. Conspicuous immunofluorescent nuclei were observed. Scale bar is 100 μm. Significant green fluorescence can be seen in the ORFV-positive serum group, but it has not been seen in other groups

Orf virus prepared from scab tissue samples was inoculated into sheep fetal fibroblasts. CPE was observed in the first generation in the sheep fetal fibroblasts (Fig. 1B). Round and swollen cells were observed after 24 h post-ORFV injection. The CPE reached 90% after 72 h, and cell shrinkage and increased intercellular space were observed, while the cells in the control group showed a tight distribution. ORFV was determined via immunofluorescence assay by comparing cross-reactions with other pathogens from positive serum, including FMDV, GTPV, and LSDV. The results showed that the nuclei of the GTPV-positive serum group, FMDV-positive serum, LSDV-positive group, and the control group were clear blue with no green fluorescence. In contrast, the ORFV-positive serum group had a noticeable green fluorescence around the nucleus (Fig. 2). In conclusion, specific immunofluorescence was observed in the ORFV-positive serum groups, which confirmed the presence of Orf viruses. The ORFV strain isolated from sheep from Sichuan, China, was named ORFV-SC and then inoculated in sheep fetal fibroblasts for passaging. The ORFV-SC strain that was attenuated by passaging was named ORFV-SC1. The passage in vitro to the 60th generation, with the increase of the virus generation, its adaptability to the cells continued to improve. After virus inoculation, the cell detoxification time was gradually shortened from 96 to 48 h, and the virus titer gradually increased from 10^3.25^ TCID_50_/ml at the 5th passage to 10^5.5^ TCID_50_/ml at the 60th passage.

### Genomic features of ORFV-SC and ORFV-SC1

The ORFV-SC isolates and ORFV-SC1 strain sequences were assembled into two contiguous sequences of 140,707 bp with 63% G + C content and 141,154 bp with 63.9% G + C content, respectively. The whole genomes of the ORFV-SC and ORFV-SC1 sequences are longer than other ORFV sequences published online (Table 1). Multiple mechanisms can affect the length of ORFV sequences, such as gene insertion or homologous recombination (Iengar 2012; Ball 1987). The coding potential of ORFV-SC and ORFV-SC1 was predicted using GATU software, and gene function annotation was performed for ORFV-SC and ORFV-SC1 by referring to PPV genomes in Table 1, 130 and 131 genes obtained for each of the two sequences. ORF002, ORF003, ORF004, ORF006, and ORF133 genes were not used in ORFV-SC and ORFV-SC1, since ORFV-SC 102 gene identity percent was lower than the threshold, the ORF102 was also not used in the ORFV-SC isolate. The ORFV-SC and ORFV-SC1 genomes also included two newly identified genes (12.5 and 107.5), proposed by Mercer et al. (Mercer et al. 2006), which had similarities with well-defined proteins in the other ORFVs, as shown in Supplemental Tables S1 and S2. Many genes in ORFV-SC and ORFV-SC1 were comparable to well-defined genes by identifying the nucleotide identities of each ORF, as shown in Supplemental Table S3. Compared to the other five ORFV isolates (NA1/11, NA17, NZ2, SY17, and CL18), ORFV-SC shared < 85% and 85–95% nucleotide identities with 10–11 genes and 10–16 genes, respectively (Table 2). The ORFV-SC and NA1/11, and SY17 shared > 95% nucleotide identity with 109 genes. In contrast, ORFV-SC1 and NA1/11 shared > 95% nucleotide identity with 109 genes and shared with 85–95% and < 85% nucleotide identity with 12–18 and 8–10 genes with the other five ORFV isolates, respectively (Table 3). The results suggested a close relationship between NA1/11 and the two sequences identified in this study.

**Table 2:** Gene number of above 95% and 85–95% nucleotide identity of ORFV-SC compared with other five ORFV isolates

ORFV-SC	% ID (aa)						
	Above 95%		85–95%		Below 85%		
	Numbers	ORFs	Numbers	ORFs	Numbers		ORFs
ORFV-NA1/11	109	001, 010, 011, 012,… 129, 130, 134	10	008, 020, 104, 107.5, 111, 118, 119, 120, 121, 134	11	005,007,009, 080, 103, 109, 110, 112, 115, 116, 132	
ORFV-NZ2	106	001, 010, 011, 012 … 129, 130, 134	14	008, 016, 020, 024, 104, 108, 111, 113, 115, 119, 120, 121, 127, 131	10	005,007,009, 080, 109, 110, 112, 116, 118, 132	
ORFV-CL18	104	010, 011, 012, 013 … 128, 129, 130	16	001, 008, 020, 024, 078, 096, 104, 107.5, 111, 113, 115, 118, 120, 121, 131, 134	10	005,007,009, 080, 103, 109, 110, 112, 116, 132	
ORFV-SY17	109	001, 010, 011, 012 … 129, 130, 134	11	008, 020, 104, 111, 113, 115, 118, 120, 121, 124, 131	10	005,007,009, 080, 103, 109, 110, 112, 116, 132	
ORFV-NA17	105	008, 010, 011, 012 … 128, 129, 130	14	001, 020, 024, 039, 061, 078, 088, 096, 107.5, 115, 119, 120, 131, 134	11	005,007,009, 080, 103, 104, 109, 110, 111, 112, 132	
ORFV-SC1	126	001, 005, 007, 008, … 131, 132, 134	1	020	3	080, 112, 116

**Table 3:** Gene number of above 95% and 85–95% nucleotide identity of ORFV-SC1 compared with other five ORFV isolates

ORFV-SC1	%ID (aa)					
	Above 95%		85–95%		Below85%	
	Numbers	ORFs	Numbers	ORFs	Numbers	ORFs
ORFV-NA1/11	109	001, 010, 011, 012, 012.5 … 130, 134	12	008, 020, 059, 080, 104, 107.5, 111, 112, 119, 120, 121	9	005, 007, 009, 103, 109, 110, 115, 116, 132
ORFV-NZ2	106	001, 010, 011 … 129, 130, 134	17	008, 016, 020, 024, 059, 080, 104, 108, 111, 112, 113, 115, 119, 120, 121, 127, 131	8	005, 007, 009, 109, 110, 116, 118, 132
ORFV-CL18	104	010, 011, 012 … 128, 129, 130	18	001, 008, 020, 024, 059, 078, 096, 104, 107.5, 111, 112, 113, 115, 118, 120, 121, 131, 134	9	005, 007, 009, 080, 103, 109, 110, 116, 132
ORFV-SY17	108	001, 010, 011, 012 … 129, 130, 134	14	008, 020, 059, 060, 104, 111, 112, 113, 115, 118, 120, 121, 124, 131	9	005, 007, 009, 080, 103, 109, 110, 116, 132
ORFV-NA17	104	008, 010, 011, 012 … 128, 129, 130	16	001, 020, 039, 059, 061, 078, 080, 088, 096, 107.5, 112, 115, 119, 120, 131, 134	10	005, 007, 009, 103, 104, 109, 110, 111, 116, 132
ORFV-SC	126	001, 005, 007, 008, … 131, 132, 134	1	020	3	080, 112, 116

### Comparison of ORFV-SC1 attenuated strains and wild ORFV-SC strains

ORFV-SC1 open reading frames were aligned with ORFV-SC isolate; MEGA 7 software was used to align each gene of SC and SC1, and the results showed that the identity of most ORFs is more than 95%, based on nucleotide sequence analysis, and some ORFs are less than 85% (Tables 2 and 3 and Supplementary information S3). Among ORFs with low identity, five ORFs (007, 020, 080, 112, 116) from SC and SC1 were analyzed further based on amino acid sequence alignment. The homology of ORF007 and ORF020 share > 85%, 86%, and 87%, respectively, and ORF080 and ORF112 share 75.2% and 75.1% identity, much greater than ORF116 share 59% identity (Supplementary Fig. S1 A). The ORF116 gene of ORFV HN3/12, NA17, SY17, and YX strains also shared < 65% amino acid identity with other published ORFV strains, suggesting that the ORF116 gene is prone to hypermutagenicity during ORFV genetic evolution (Chen et al. 2017; Chi et al. 2015; Zhong et al. 2019).

### Effect of mutation on protein structure

**Fig. 3:**
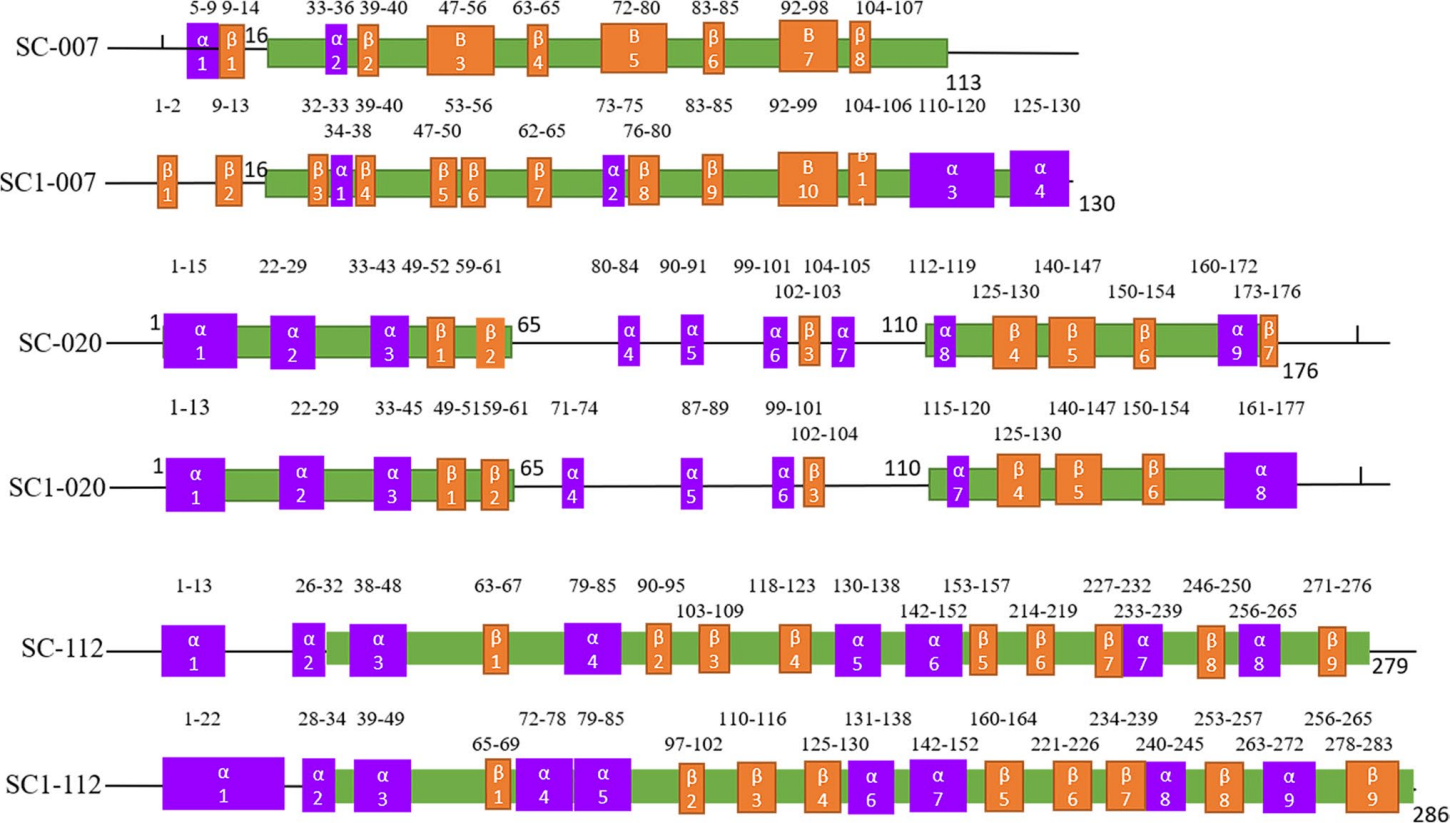
Secondary structure and functional domain analysis of ORF007, ORF020, and ORF112 Proteins between ORFV-SC and ORFV-SC1 strains. Green: functional domain; purple: alpha helices structure (α); yellow: beta folds structure (β)

Protein functional domains and secondary structures of ORF007, ORF020, and ORF112 from SC and SC1 isolates were analyzed using SMART and SOPMA web platforms which are shown in Supplementary Tables S4:1–6 and S5:1–6 and Table 4. The results indicated that the ORF007, ORF020, and ORF112 functional domains are essentially identical between SC and SC1 strains, except that the SC1-007 functional domain is located at 16–130 while SC-007 is 16–113. The secondary structure analysis shows that the main structure of the ORF007 protein was the beta turns and random coils, which did not change much between the SC and SC1 strains. The number of alpha helices(α) went up from 7.08 to 19.23%, while the proportion of beta folds (β) went down by 10%. The ratio of the four structures in ORF020 and ORF112 proteins does not change much between SC and SC1 isolates, and the main structures are the alpha helix and random coil, as shown in Table 4. To further investigate the effect of mutations on the secondary structure of these three proteins, based on the alpha helix, beta fold, and functional domain mapping, the results revealed that SC1-ORF007 had two additional alpha helix and three beta fold structures, some of which had positions changed. Compared to SC-ORF020, the number of alpha helix and beta fold structures in SC1-ORF020 altered, with α4, α5, α7, and α8 positions changing; α2, α3, α4, α5, and α6 remained mostly constant in ORF112, but other alpha helix and beta fold structure locations changed due to mutations (positions α and β refer to SC strains), as shown in Fig. 3.

**Table 4:** The result of ORFV007, ORFV020, and ORFV112 protein structure analysis

Project name	Protein					
	SC-007	SC1-007	SC-020	SC1-020	SC-112	SC1-112
Size (aa)	113	130	183	183	280	287
Secondary structure (%)						
Alpha helix	8/113 (7.08%)	25/130 (19.23%)	68/183 (37.26%)	68/183 (37.16%)	77/280 (27.50%)	89/287 (31.01%)
Extended strand (beta fold)	47/113 (41.59%)	41/130 (31.54%)	35/183 (19.13%)	33/183 (18.03%)	54/280 (19.29%)	54/287 (18.82%)
Beta turn	18/113 (15.93%)	16/130 (12.31%)	11/183 (6.01%)	9/183 (4.92%)	15/280 (5.36%)	16/287 (5.57%)
Random coil	40/113 (35.40%)	48/130 (36.92%)	69/183 (37.70%)	73/183 (39.89%)	134/280 (47.86%)	128/287 (44.60%)
Domain (position)	16–113	16–130	1–65 110–176	1–65 110–176	32–279	33–286
Tertiary structure I-TASSER						
C-score	0.51	0.76	− 3.25	− 1.79	− 0.39	− 0.29
TM-score	0.78 ± 0.10	0.82 ± 0.09	0.35 ± 0.12	0.50 ± 0.15	0.66 ± 0.13	0.68 ± 0.12
RMSD	3.2 ± 2.3 Å	3.0 ± 2.1 Å	12.7 ± 4.2 Å	9.1 ± 4.6 Å	6.9 ± 4.1 Å	6.7 ± 4.0 Å

**Fig. 4:**
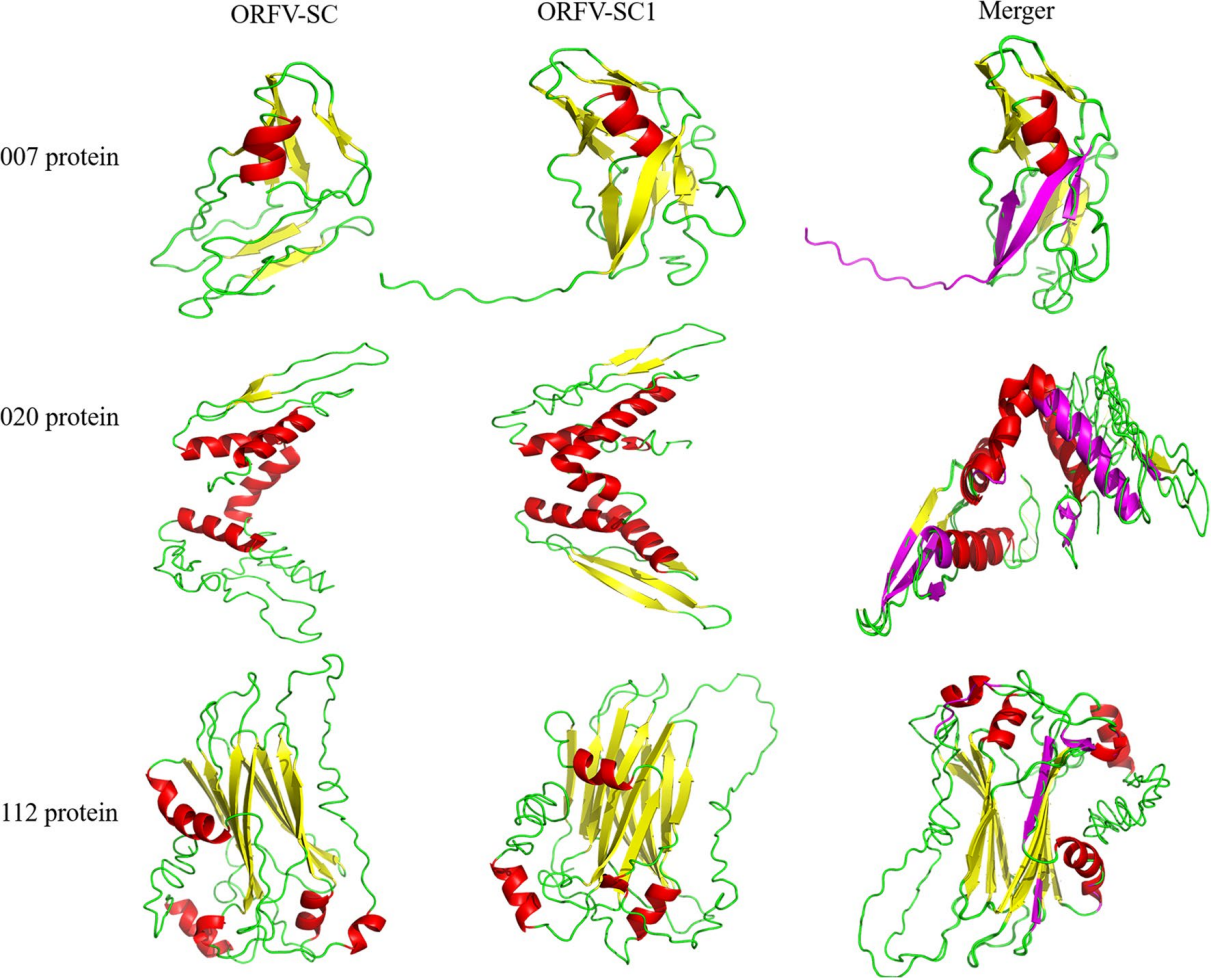
Tertiary structure analysis of ORF007, ORF020, and ORF112 proteins between ORFV-SC and ORFV-SC1 isolate. Red: helix structure; yellow: sheet structure; green: loop structure; magentas: difference structures

The crystal protein structure of ORF007, ORF020, and ORF112 between SC and SC1s strains was predicted by the powerful online webserver I-TASSER. Referring to the TM-score and RMSD values, the protein tertiary structure with the highest C-score was selected from the final five prediction models, the result shown in Supplementary Table S6:1–6 and Table 4. Predicted 3D structures were analyzed using pyMOL software, while differences in the tertiary structure of SC and SC1 proteins were compared using align function. In the SC1 strain, the ORF007 protein gains three sheet structures, the ORF020 protein loses four loop structures while gaining two helix structures and four sheet structures, and the ORF112 protein undergoes a major transition from helix to loop structure (Fig. 4). Based on the information above, mutations in the ORF007, ORF020, and ORF112 genes of the SC and SC1 strains change the secondary structure and crystal structure of proteins but have no effect on their functional domains.

### Phylogenetic analysis

**Fig. 5:**
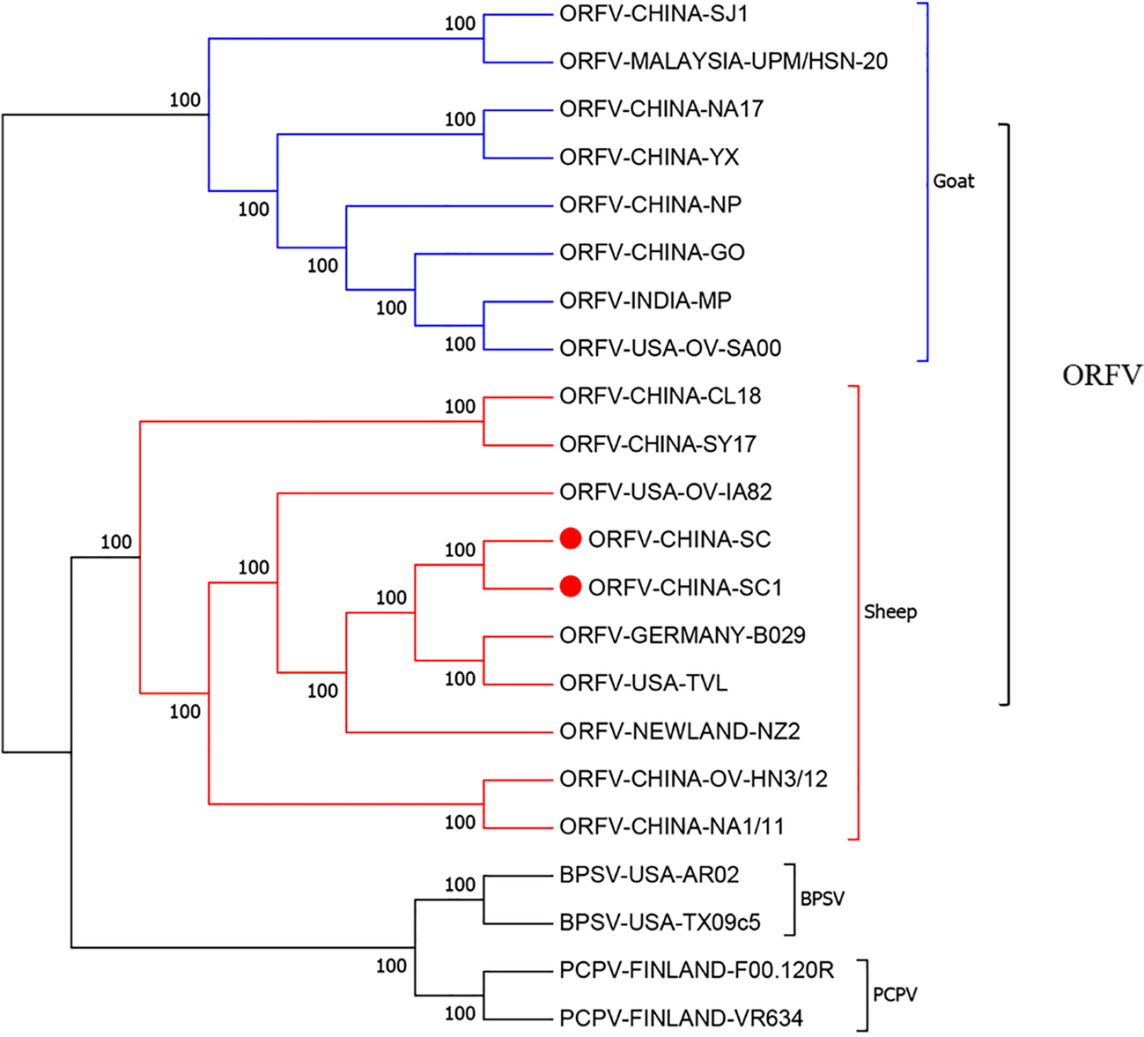
Phylogenetic analyses based on whole genome sequences of PPVs. The phylogenetic tree was constructed by the maximum-likelihood method using IQ-TREE software. The numbers above or below the branch points indicate the bootstrap support calculated for 1000 replicates

**Fig. 6:**
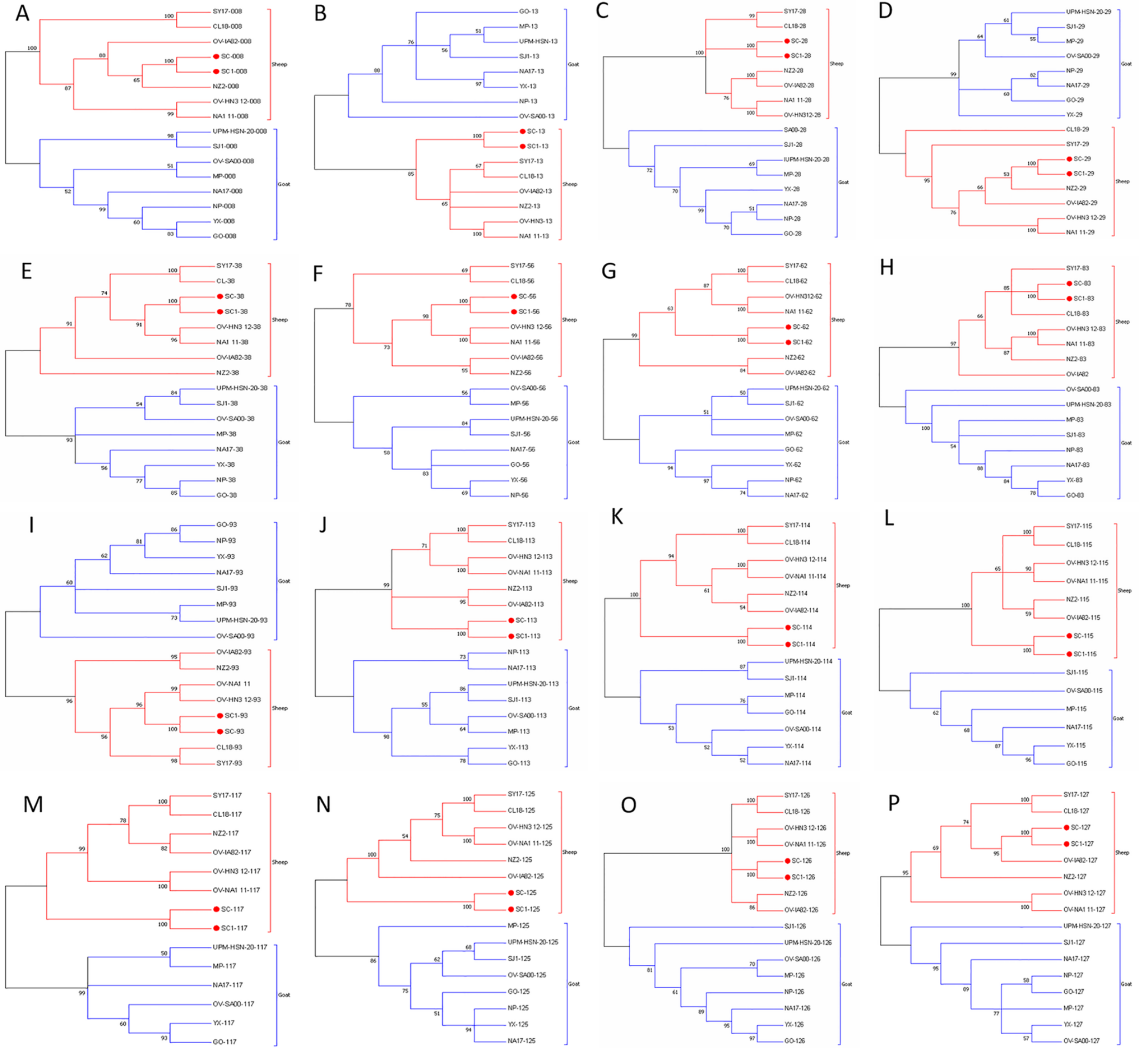
Phylogenetic analysis based on the nucleotide sequence of ORFV single gene distinguishing sheep and goat origination. The phylogenetic relation was constructed by the maximum-likelihood method using IQ-TREE software. Numbers at the branching points indicate the bootstrap support calculated for 1000 replicates: (**A**) ORFV008, (**B**) ORFV013, (**C**) ORFV028, (**D**) ORFV029, (**E**) ORFV030, (**F**) ORFV056, (**G**) ORFV062, (**H**) ORFV083, (**I**) ORFV093, (**J**) ORFV113, (**K**) ORFV114, (**L**) ORFV115, (**M**) ORFV117, (**N**) ORFV125, (**O**) ORFV126, and (**P**) ORFV127

Phylogenetic analysis based on the complete genomic sequences of 22 PPVs revealed that eight ORFV isolates originated in goats and ten ORFV isolates originated in sheep, which formed two separate branches with 100% bootstrap support (Fig. 5). ORFV-SC and ORFV-SC1 isolated from sheep showed a close relationship with ORFV-B029 and ORFV-TVL, although ORFV-B029 is a human infection strain originating from sheep ORFV. To investigate the genetic characteristics and phylogenetic relationships, we further analyzed the phylogenetic trees of each gene (128 in total) sequence of ORFVs. Phylogenetic trees based on each of 37 ORFs (007, 008, 013, 019, 020, 028, 029, 038, 041, 043, 049, 050, 051, 056, 061, 062, 063, 073, 083, 088, 093, 113, 114, 115, 116, 117, 118, 120, 121, 122, 125, 126, 127, 128, 129, 130, and 134) with greater than 50% bootstrap support showed that ORFVs originating from goats and sheep formed separate branches, suggesting ORFV-SC and ORFV-SC1 mostly originate from sheep. Sixteen of those 37 ORFs (008, 013, 028, 029, 038, 056, 062, 083, 093, 113, 114, 115, 117, 125, 126, and 127) with bootstrap values greater than 70% at the node between goat branch and sheep branch and bootstrap values great than 50% at all nodes in goat branch and in sheep branch are summarized in Fig. 6, while the other 21 genes are summarized in Supplementary Figs. S2 and S3. The other genes (91 in total) lack phylogenetic signal to separate goat from sheep origins, and most of them cannot form separate goat origination and sheep origination branches, while some ORFs (009, 010, 014, 059, 081, and 098) have a phylogenetic signal to separate goat from sheep origins but with very low bootstraps (Supplementary Figs. S4, S5, S6, S7, S8, and S9). The analysis results indicated that the genetic relationship of ORFV strains derived from the same host species was closer.

### Mild pathological changes in various tissues caused by the attenuated ORFV-SC1 strain inoculation in rabbits

**Fig. 7:**
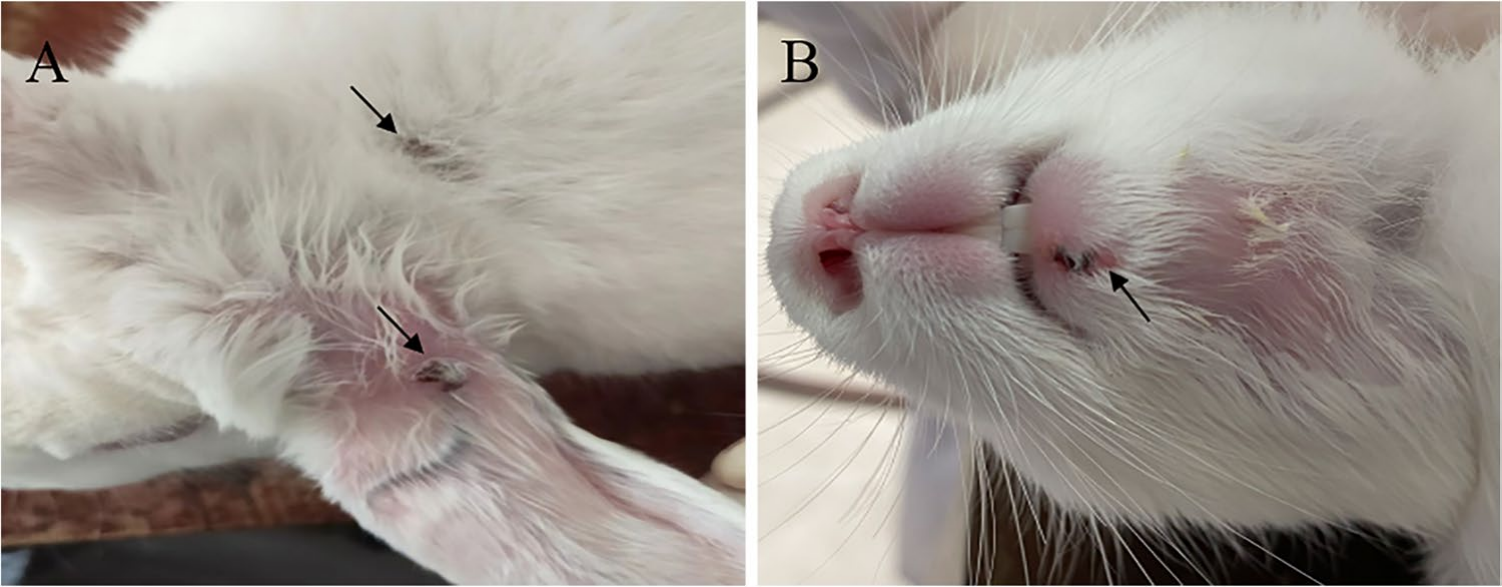
Typical clinical symptoms produced by ORFV-SC inoculation of rabbit orally. (**A**) Rabbits inoculated orally with ORFV-SC developed scabs on the ear margin lips and neck on day 7. (**B**) Rabbits inoculated orally with ORFV-SC developed scabs on the lips on day 7

**Fig. 8:**
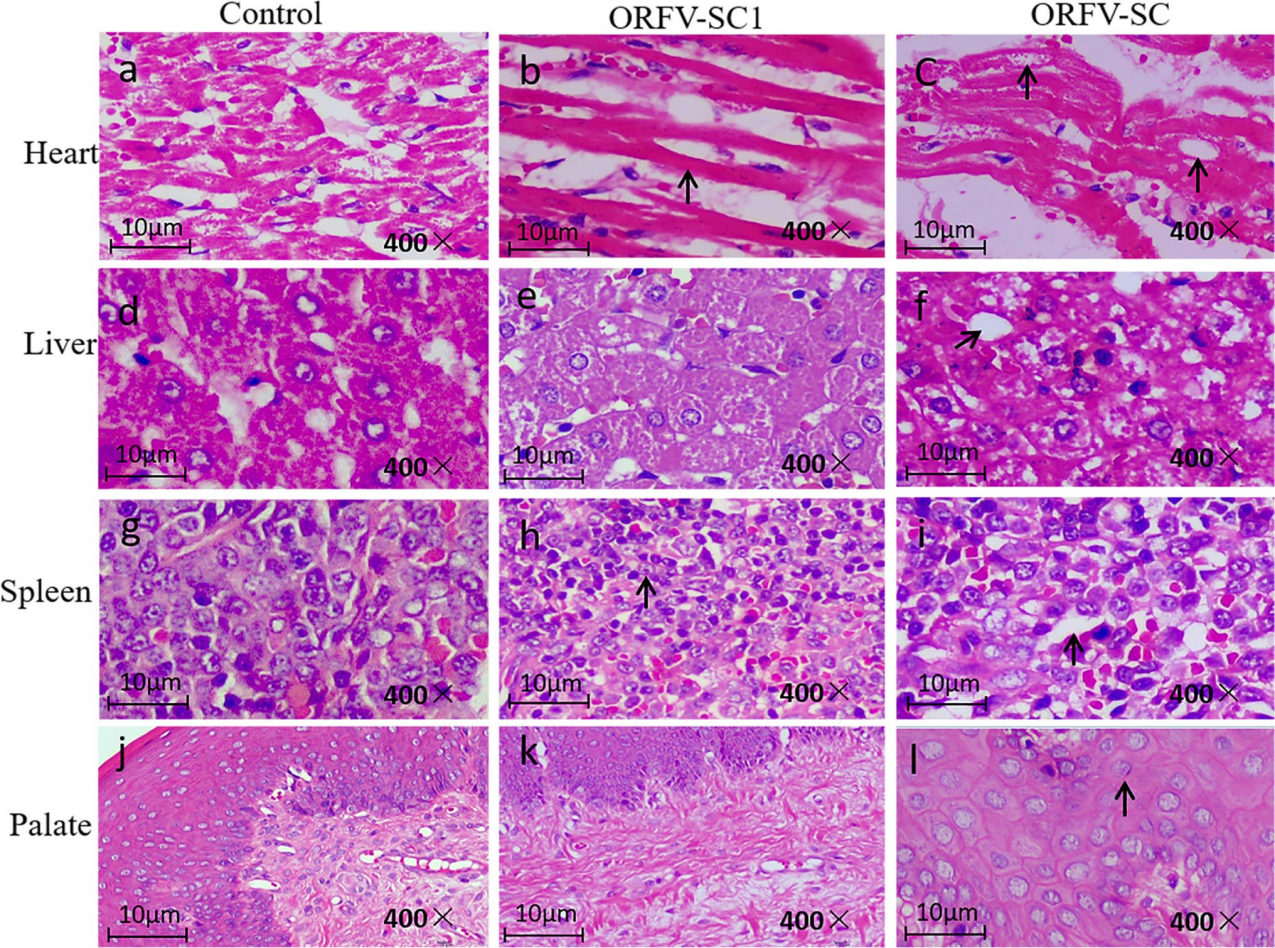
Pathological changes in the tissues of rabbits infected with ORFV-SC and ORFV-SC1. HE staining of histopathological changes (left to right in each row) in the heart, liver, spleen, and palate of rabbits inoculated with ORFV-SC1 and ORFV-SC, respectively

**Fig. 9:**
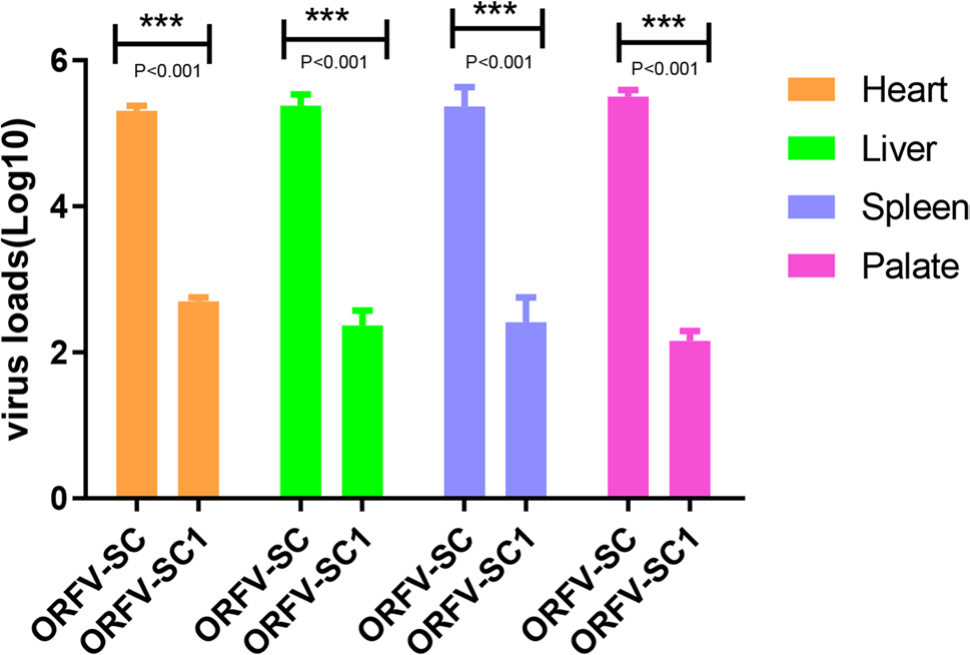
Differences in viral load between tissues. The virus loads in the heart, liver, spleen, and palate were significantly different between the two groups

After inoculation, the rabbits were monitored every day, observing for surface characteristics and measuring body temperature. Rabbit tissues were collected and fixed in 4% paraformaldehyde on day 7, and pathological sections were prepared. Three rabbits in group 1 (inoculated with ORFV-SC wild virus) showed scabs at the chin, ear margin, and neck at about day 7, which lasted 3–7 days (Fig. 7). In contrast, group 2 (vaccinated with ORFV-SC1 attenuated strain) rabbits did not develop any lesions on the body surface (not show). In addition, there was no significant difference in body temperature between the two groups of rabbits, which was the same as the control group. Rabbits in group 2 had better feed intake, while some rabbits in group 1 demonstrated malaise on days 7–14 and then gradually returned to normal. To compare the damage of wild ORFV-SC isolate and attenuated ORFV-SC1 to animal health by evaluating the degree of damage to various tissues after infection with wild SC isolate and attenuated SC1 strain (Fig. 8). In the control group, the structure of the epicardium was intact and clear; the myocardial fibers in the medial layer were arranged neatly, whereas the myocardial fibers of rabbits in group 2 became thinner. No other pathological changes were observed. In contrast, the myocardial fibers of rabbits in group 1 showed degeneration and necrosis, with the dissolution of necrotic myocardial fibers, degeneration of cytoplasmic granules, accompanied by vacuoles, and a small amount of cytoplasmic dissolution (Fig. 8a–c). Compared with the liver of the control group, no obvious pathological changes were observed in the hepatocytes of rabbits in group 2. However, a small amount of vacuolar degeneration was observed in the hepatocytes of group 1 rabbits, with vacuoles of different sizes in the cytoplasm and suspended nuclei in the center of the cells or located on one side (Fig. 8d–f). Decreased splenic lymphocytes, white pulp atrophy, and marginal zone loss were observed in group 2 rabbits. In group 1 rabbits, the splenic white and red pulp demarcation was not clear, and a significant decrease in lymphocytes, marginal zone loss, activation of the red pulp reticular system, increased reticular cells, and large sinusoids were observed (Fig. 8g–i). Compared with the control group, only mucosal epithelial thickening and disarrangement were found in the palate of rabbits in both groups. The loose connective tissue in the submucosa contained palatal glands, blood vessels, lymphatic vessels, tonsils, and crypts (Fig. 8j–l). Moreover, no significant lesions were found in the lungs, kidneys, lips, and other tissues. Viral load was detected by RT-qPCR in tissues with pathological changes to evaluate the viral load in various organs of rabbits infected with the wild isolate ORFV-SC or with the cell passage strain ORFV-SC1 (Fig. 9). RT-qPCR confirmed the reliability of the pathological section results. In conclusion, attenuated ORFV-SC1 strains are less damaging to animals than wild virus ORFV-SC. In addition, animals infected with attenuated viruses recovered rapidly and showed modest clinical signs, indicating that the ORFV-SC1 strain has the potential to serve as a vaccine.

## Discussion

Orf is an acute, epitheliotropic human-veterinary co-infectious disease caused by the Orf virus (ORFV) that commonly affects sheep and goats. The disease is highly contagious, has a high incidence, and is distributed globally. It causes significant economic losses to the breeding industry while seriously endangering public health and safety. Due to the low morbidity rates, ORFV infection in sheep and goats is frequently overlooked. Although many ORFV outbreaks have been documented in China (Chen et al. 2017; Chi et al. 2015; Hua et al. 2015; McKenna et al. 2010), ORFV receives far less attention than other illnesses such as sheep pox, pest des petits ruminants, and foot-and-mouth disease.

One ORFV strain was successfully isolated from diseased sheep in China’s Sichuan region and was named ORFV-SC (GenBank accession number ON932451.1). This condition is characterized by lesions around the lips, muzzle, and nostrils (Fig. 1a,b). Immunofluorescence techniques were used to confirm the diagnosis. The immunofluorescence results confirmed ORFV infection and that no additional viruses were present. The ORFV-SC isolate was then passaged in sheep fetal fibroblasts to the 60th generation strain, yielding the ORFV-SC1 strain (GenBank accession number ON932452.1). Whole genome sequencing and analysis of ORFV-SC and ORFV-SC1 strains were performed to determine whether ORFV-SC1 strains can be applied as attenuated vaccination. Furthermore, the findings of this study provide a better understanding of ORFV virulence, disease pathogenesis, and genome sequence diversity among isolates.

ORFV-SC (140707 bp) and ORFV-SC1 (141154 bp) genomic sequences were collected and analyzed using comparative genomics. The sequences obtained in this report exceed the longest previously reported ORFV-SY17 (140,413 bp) (Zhong et al. 2019). The genome structure of ORFV in this study was similar to that of other reference ORFV strains, with 130 and 131 predicted genes, 63% and 63.9% G + C content, respectively. After ORF comparison among ORFVs, a large core coding region (ORFV009-111) encoding relatively conserved genes was found, with both ends being inverted terminal repeats (ORFV001-008 and ORFV112-134) with many mutations. These results were similar to other studies (Tsai et al. 2009; Li et al. 2015). The nucleotide homology results demonstrated a close association between SC, SC1, and NA1/11, which may be related to their origin in the same host. To compare the variation between wild strains and attenuated strains passaged to 60 generations and to explore the causes of attenuated strain weakening, multiple alignments of the nucleotide and amino acid sequences of the ORFs of ORFV-SC and ORFV-SC1 strains revealed mutations in ORF007, ORF020, ORFV080, ORF112, and ORF116 caused by insertions or deletions, and mutations in these amino acids caused changes in the secondary and tertiary structures of ORF007, ORF020, and ORF112 genes. ORF112 genes encode CBP protein, which is an important virulence factor, and ORFV112 deletion resulted in the attenuation of ORFV NZ2 (Fleming et al. 2017). Mutations in these proteins may be closely related to viral evolution and its self-protection mechanism, as well as viral weakening.

To examine the evolutionary tendencies of our two ORFVs and other PPVs, phylogenetic trees were generated based on complete genome sequences. ORFV isolates from goats and sheep formed distinct branches, while ORFV-SC, ORFV-SC1, and ORFV isolates from sheep formed one branch. The ORFV-TVL isolate is of the same branch and is closely related to ORFV-SC and ORFV-SC1 and is a vaccine strain obtained by serial passage in ovine testicular cells (USDA product serial number 1821.51). This result is consistent with the discovery that PPV adaptation to growth in cell lines can result in substantial genomic changes, as proven for the highly attenuated ORFV isolate D1701 (Friederichs et al. 2014). Analysis of the phylogenetic trees based on nucleotide sequences of each gene of ORFV, 37 genes in total were found to assist in easily distinguishing between goat and sheep-originated ORFVs.

In this study, the incidence, incubation time, and duration of local characteristics were compared, and pathological sections in two groups of rabbits were observed to assess the ORFV-SC1 strain virulence. Rabbits vaccinated with the attenuated strain SC1 exhibited a lower morbidity rate, no clear clinical ORFV symptoms, and considerably lower virus loads in the heart, liver, spleen, and palate than rabbits vaccinated with the virulent strain SC, resulting in less pathological alterations. Our findings differed from those of earlier research. Clinical signs were observed 7 days following ORFV-SC injection in rabbits, whereas other investigations indicated that they were visible after 3 days (Cargnelutti et al. 2011). Strain selection, inoculation location, viral infection during inoculation, and other factors could explain the difference in clinical presentation. The dosage, virus, and injection location of ORFV-SC1 and ORFV-SC were all the same. Yet, no major pathological alterations occurred compared to rabbits in the ORFV-SC group, demonstrating that ORFV-SC1 was weakened through repeated passage.

In summary, additional genetic information on ORFV-SC and ORFV-SC1 was collected from sheep in Sichuan province, southwest China. Furthermore, the present findings may give some insight into the pathogenic genotypes responsible for ORFV outbreaks in Sichuan Province. The results also provide a foundation for the future development of ORFV-attenuated vaccines.

## Supplementary Information

Below is the link to the electronic supplementary material.

Supplementary Fig. S1 (PNG 2940 kb)

High resolution image (TIF 1454 kb)

Supplementary Fig. S2 (PNG 551 kb)

High resolution image (TIF 354 kb)

Supplementary Fig. S3 (PNG 699 kb)

High resolution image (TIF 507 kb)

Supplementary Fig. S4 (PNG 2889 kb)

High resolution image (TIF 1495 kb)

Supplementary Fig. S5 (PNG 793 kb)

High resolution image (TIF 531 kb)

Supplementary Fig. S6 (PNG 763 kb)

High resolution image (TIF 522 kb)

Supplementary Fig. S7 (PNG 810 kb)

High resolution image (TIF 525 kb)

Supplementary Fig. S8 (PNG 764 kb)

High resolution image (TIF 505 kb)

Supplementary Fig. S9 (PNG 856 kb)

High resolution image (TIF 582 kb)

Supplementary Table S1 (XLSX 21 KB)

Supplementary Table S2 (XLSX 21 KB)

Supplementary Table S3 (XLSX 13 KB)

Supplementary Table S4-1 (HTML 15 KB)

Supplementary Table S4-2 (HTML 15 KB)

Supplementary Table S4-3 (HTML 17 KB)

Supplementary Table S4-4 (HTML 16 KB)

Supplementary Table S4-5 (HTML 19 KB)

Supplementary Table S4-6 (HTML 19 KB)

Supplementary Table S5-1 (HTML 23 KB)

Supplementary Table S5-2 (HTML 23 KB)

Supplementary Table S5-3 (HTML 29 KB)

Supplementary Table S5-4 (HTML 30 KB)

Supplementary Table S5-5 (HTML 25 KB)

Supplementary Table S5-6 (HTML 24 KB)

Supplementary Table S6-1 (HTML 92 KB)

Supplementary Table S6-1 (PDB 157 KB)

Supplementary Table S6-2 (PDB 132 KB)

Supplementary Table S6-2 (HTML 89 KB)

Supplementary Table S6-3 (PDB 216 KB)

Supplementary Table S6-3 (HTML 83 KB)

Supplementary Table S6-4 (HTML 86 KB)

Supplementary Table S6-4 (PDB 218 KB)

Supplementary Table S6-5 (HTML 129 KB)

Supplementary Table S6-5 (PDB 341 KB)

Supplementary Table S6-6 (HTML 126 KB)

Supplementary Table S6-6 (PDB 336 KB)

## Data Availability

The authors confirm that the data supporting the findings of this study are available within the article.
