# Correction to: The whole genomic analysis of the Orf virus strains ORFV‑SC and ORFV‑SC1 from the Sichuan province and their weak pathological response in rabbits

**DOI:** 10.1007/s10142-023-01120-1

**Published:** 2023-05-24

**Authors:** Guoyu Du, Jinyan Wu, Cheng Zhang, Xiaoan Cao, Lingxia Li, Jijun He, Yong Zhang, Youjun Shang

**Affiliations:** 1 College of Veterinary Medicine, Gansu Agricultural University, Lanzhou, 730046 China; 2 State Key Laboratory for Animal Disease Control and Prevention, Lanzhou Veterinary Research Institute, Chinese Academy of Agricultural Sciences, Lanzhou, 730046 China; 3 State Key Laboratory of Veterinary Etiological Biology, Lanzhou Institute of Veterinary Research (CAAS) Institute, Chinese Academy of Agricultural Sciences, Lanzhou, 730046 China

**Correction to: Functional & Integrative Genomics 2023**

**https://doi.org/10.1007/s10142-023-01079-z**

The online version of the article contains an error during publication. In Funding, the first project number, SQ2022YFD1600330, is the number automatically generated at the time of the project application, not the final project number. The correct project number is 2022YFD1602203.

The original article has been corrected.

