# Supplementary figures and images for: The whole genomic analysis of the Orf virus strains ORFV-SC and ORFV-SC1 from the Sichuan province and their weak pathological response in rabbits
Source: Funct Integr Genomics. 2023 May 16;23(2):163. doi: 10.1007/s10142-023-01079-z (PMC10185592; doi:10.1007/s10142-023-01079-z)

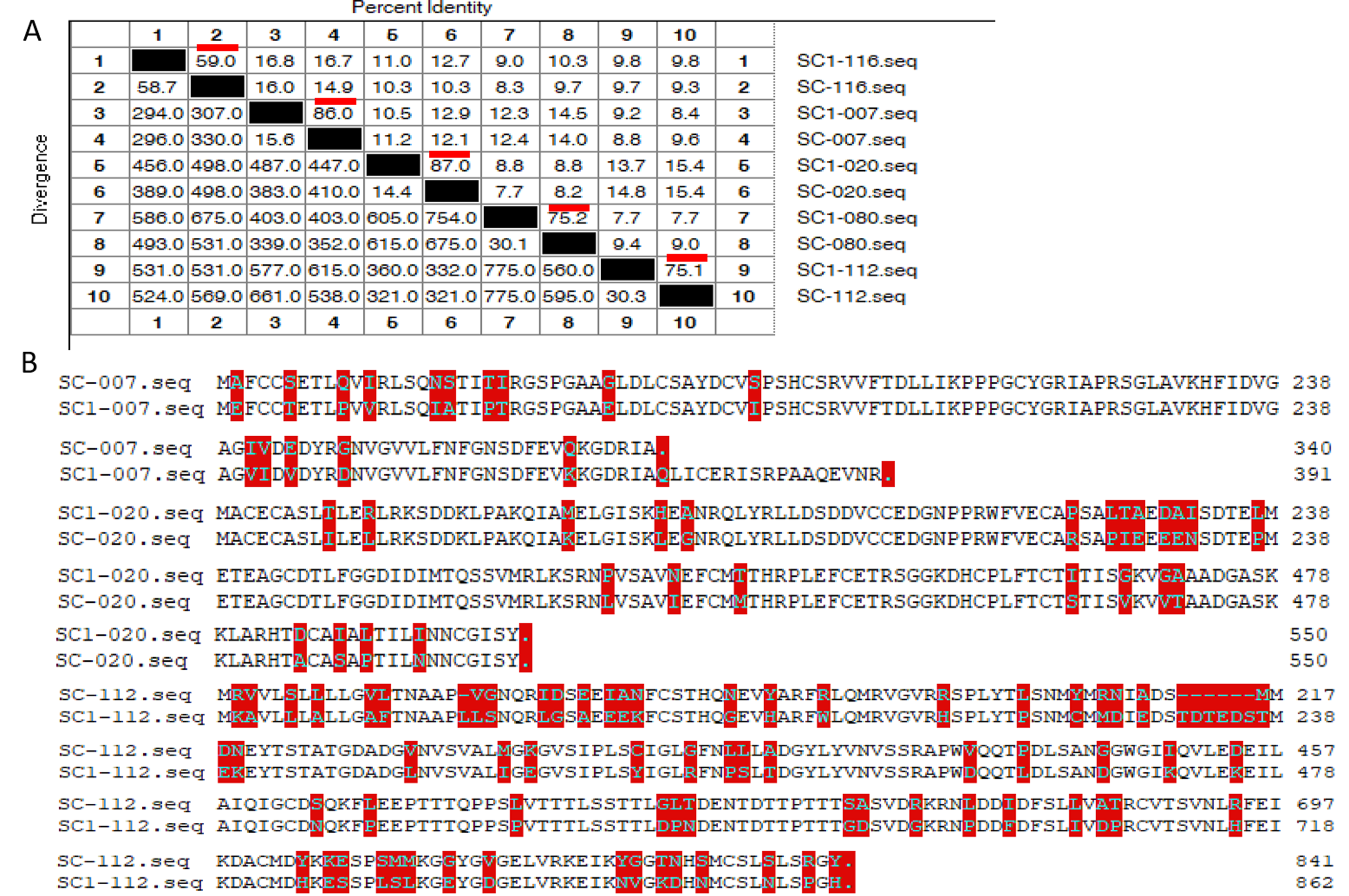

Supplement: Supplementary file 1 — (PNG 2940 kb) [file 10142_2023_1079_Fig10_ESM.png]

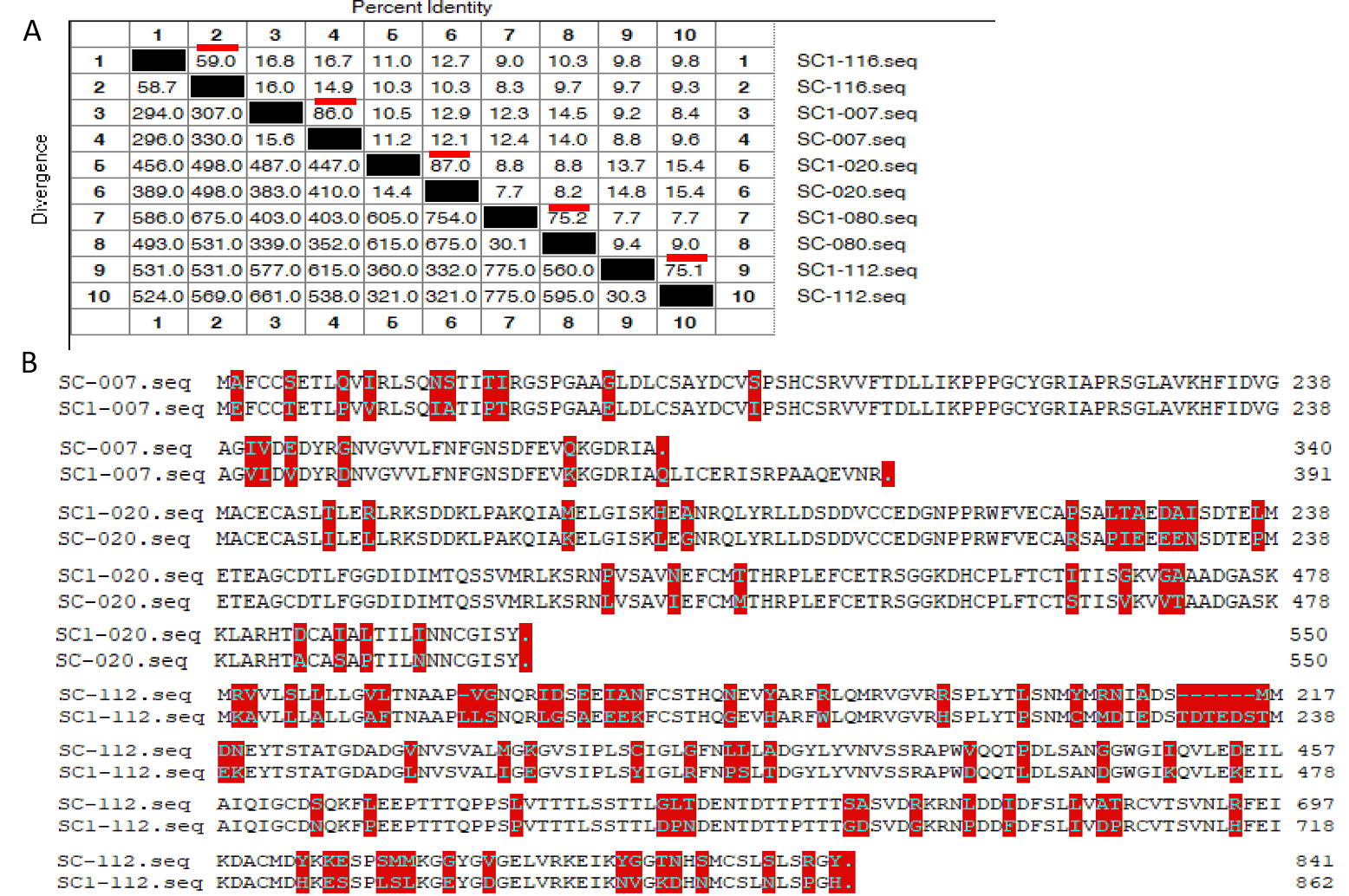

Supplement: Supplementary file 2 — High resolution image (TIF 1454 kb) [file 10142_2023_1079_MOESM1_ESM.tif]

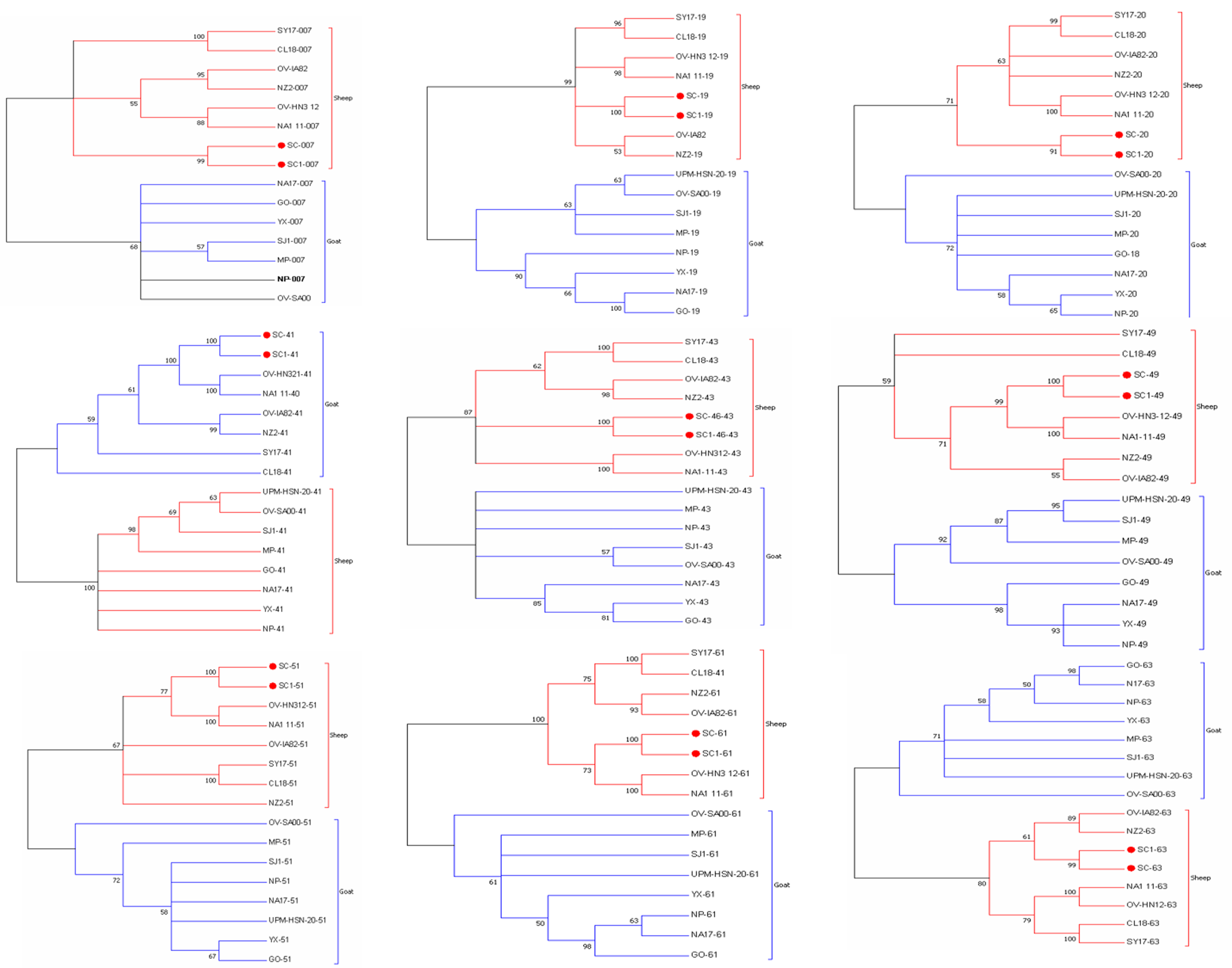

Supplement: Supplementary file 3 — (PNG 551 kb) [file 10142_2023_1079_Fig11_ESM.png]

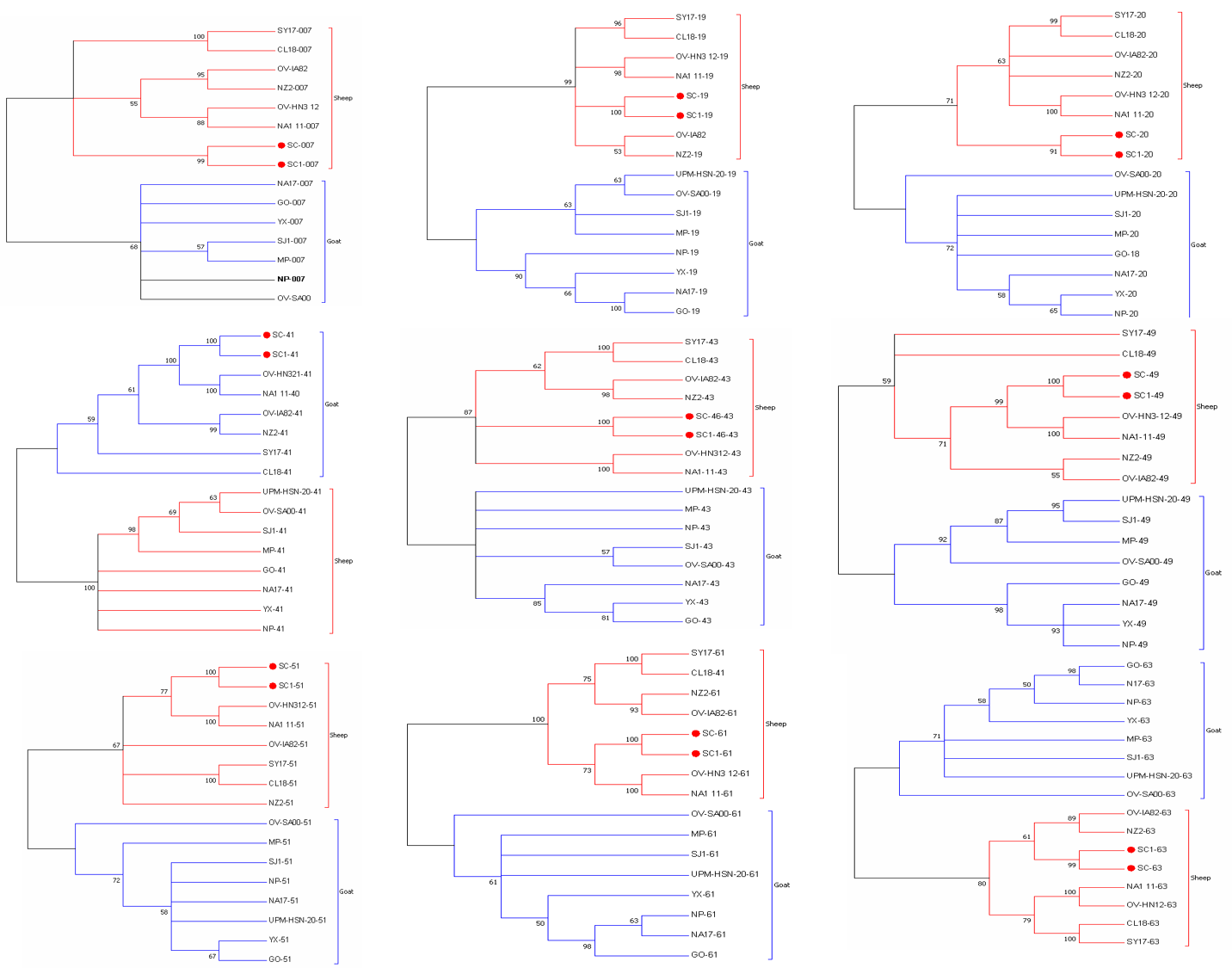

Supplement: Supplementary file 4 — High resolution image (TIF 354 kb) [file 10142_2023_1079_MOESM2_ESM.tif]

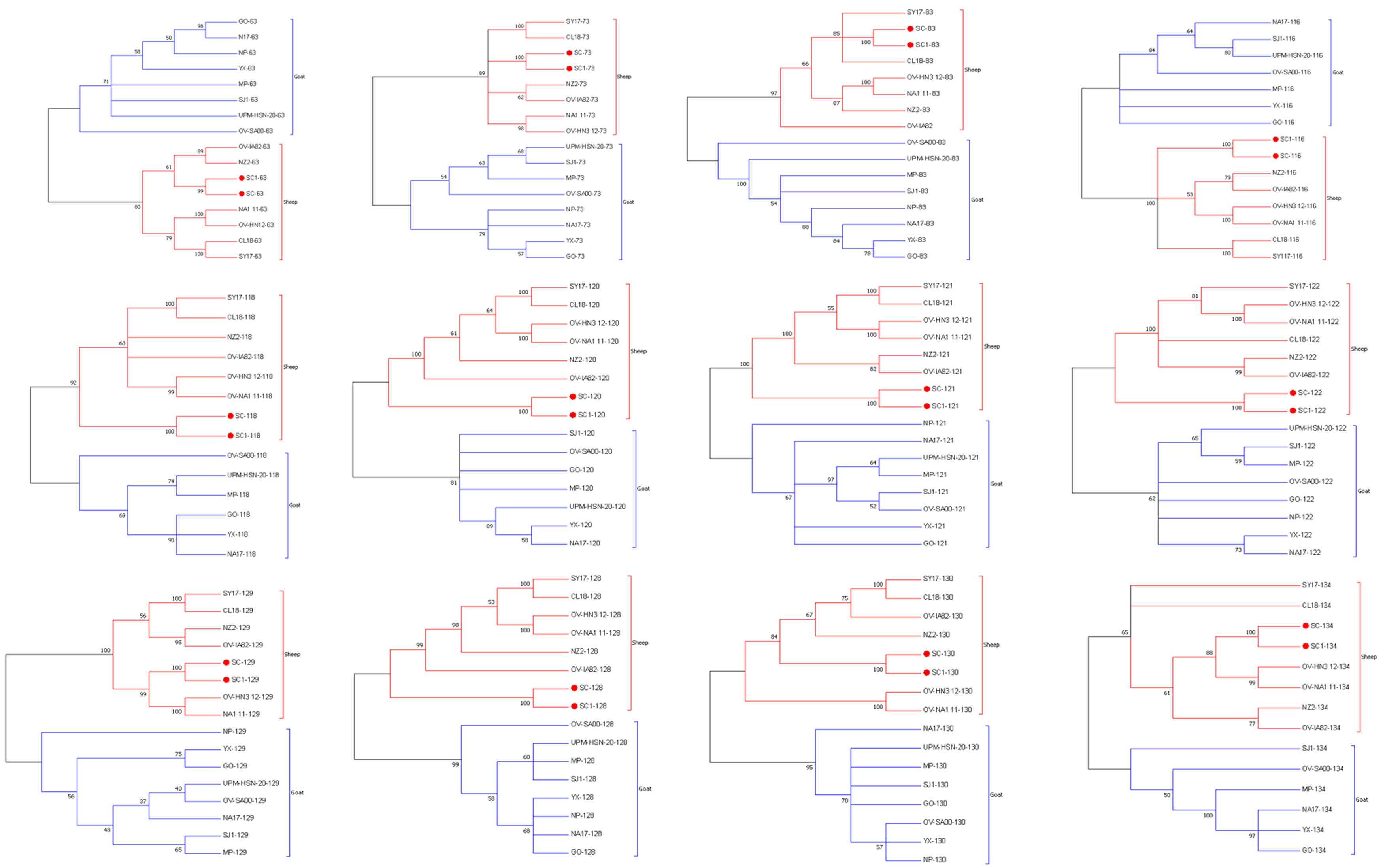

Supplement: Supplementary file 5 — (PNG 699 kb) [file 10142_2023_1079_Fig12_ESM.png]

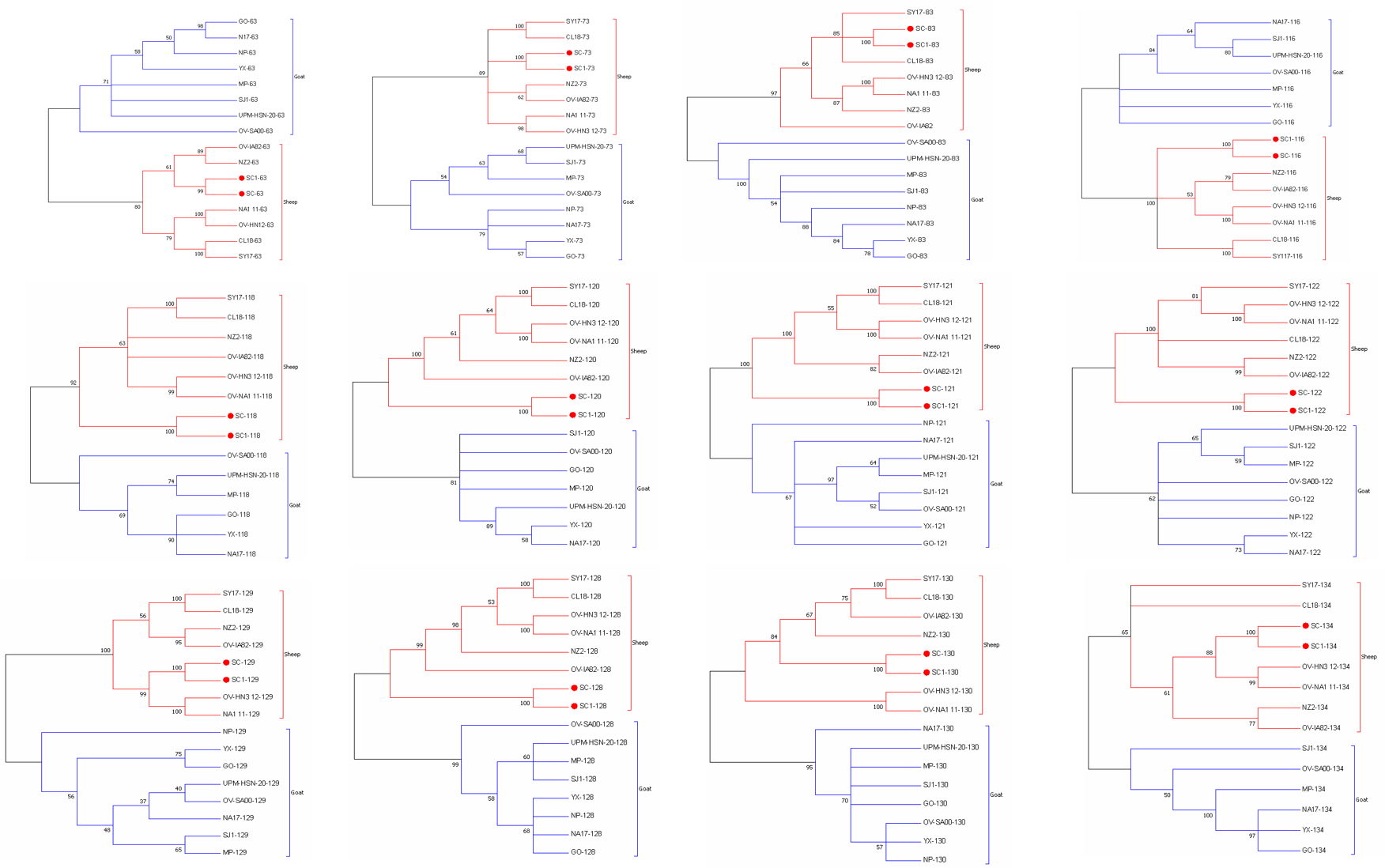

Supplement: Supplementary file 6 — High resolution image (TIF 507 kb) [file 10142_2023_1079_MOESM3_ESM.tif]

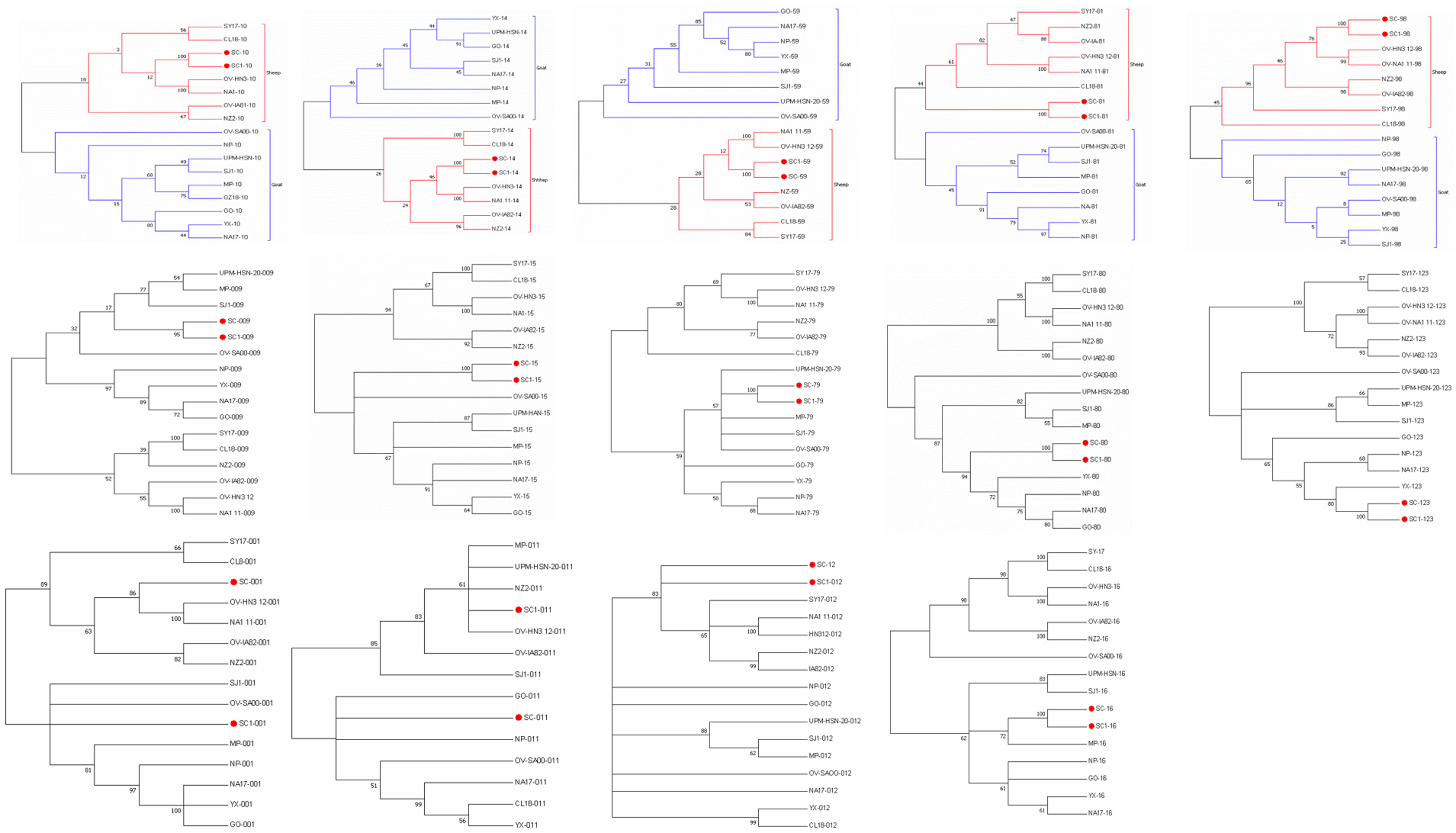

Supplement: Supplementary file 7 — (PNG 2889 kb) [file 10142_2023_1079_Fig13_ESM.png]

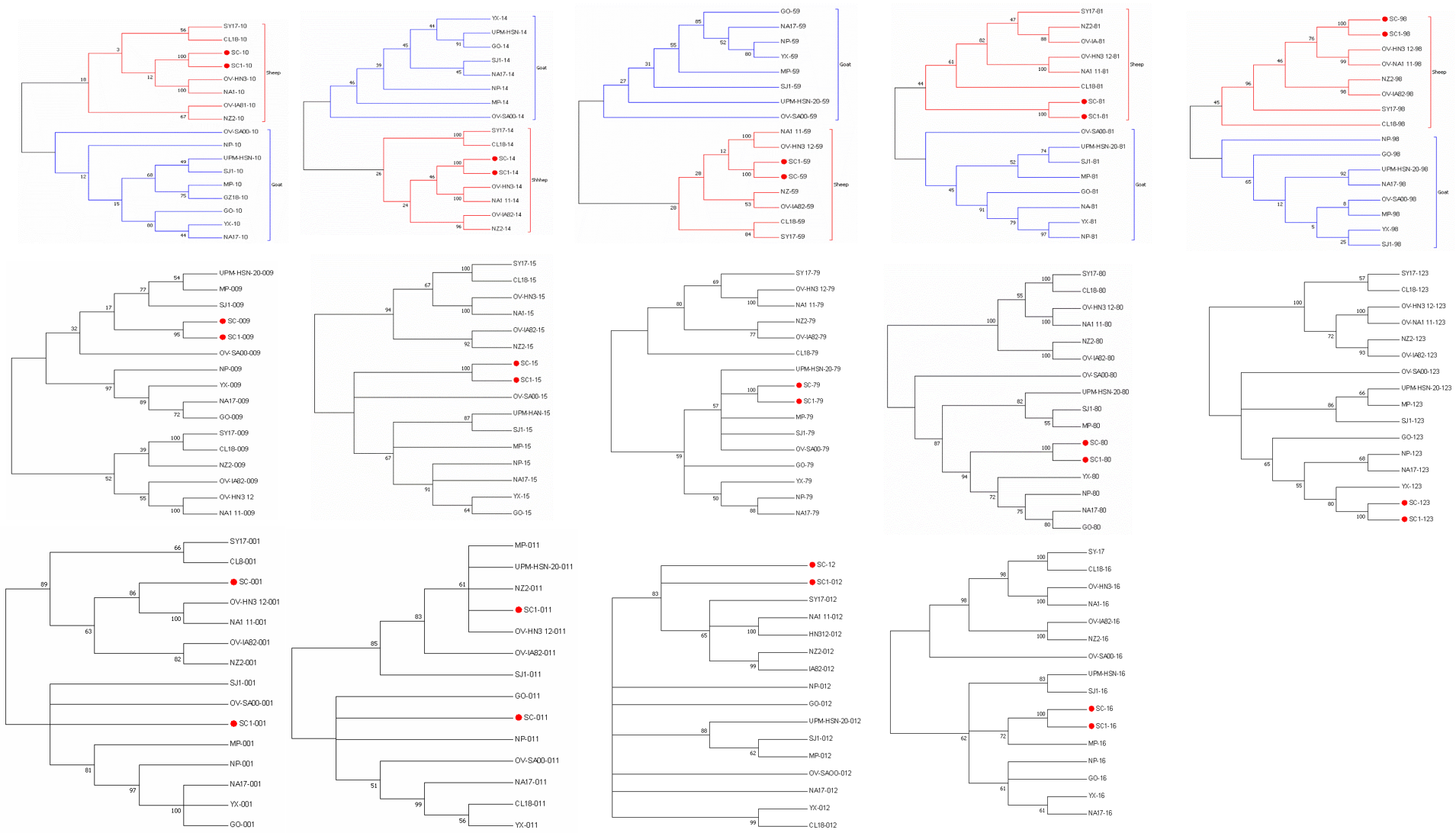

Supplement: Supplementary file 8 — High resolution image (TIF 1495 kb) [file 10142_2023_1079_MOESM4_ESM.tif]

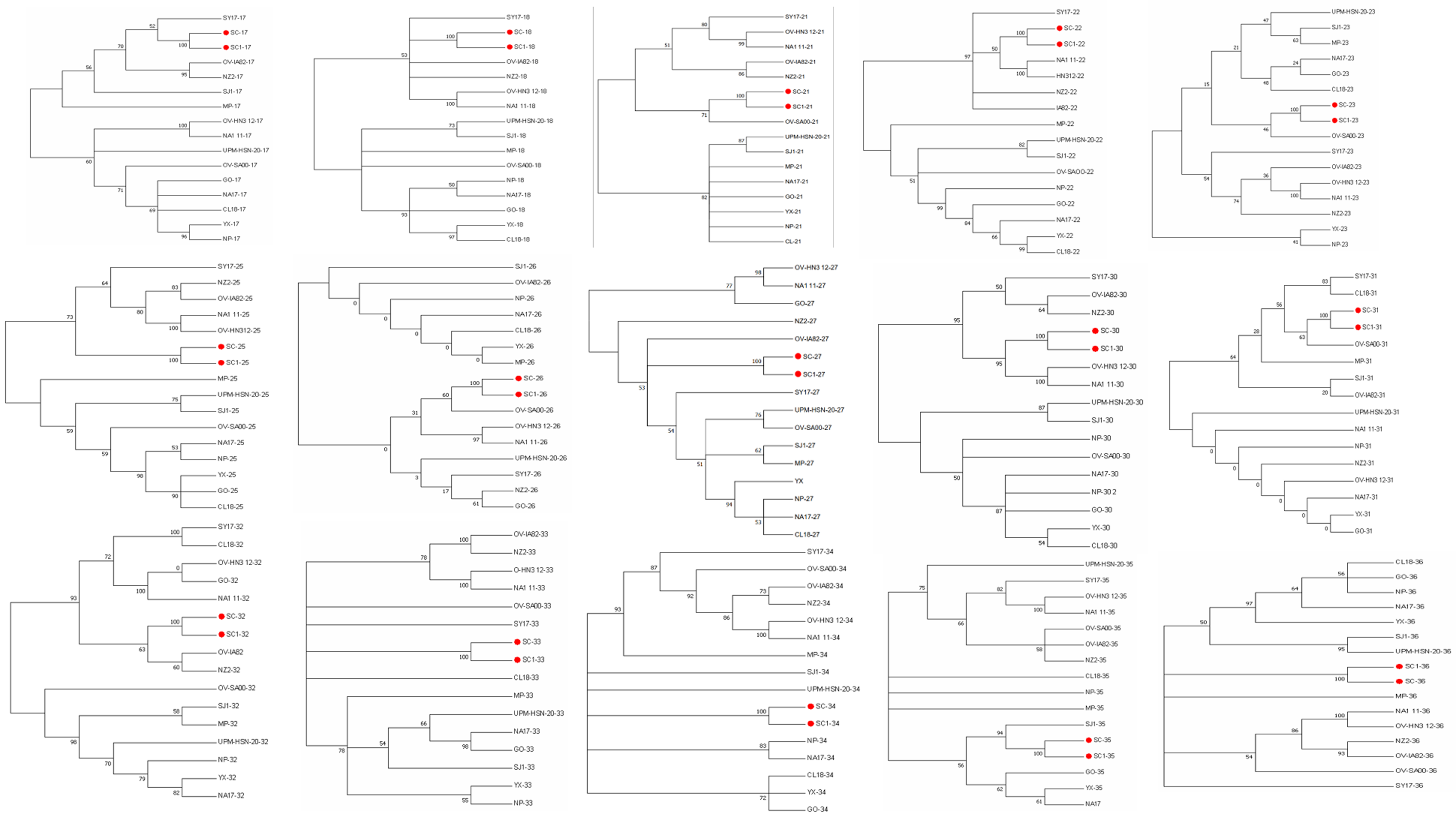

Supplement: Supplementary file 9 — (PNG 793 kb) [file 10142_2023_1079_Fig14_ESM.png]

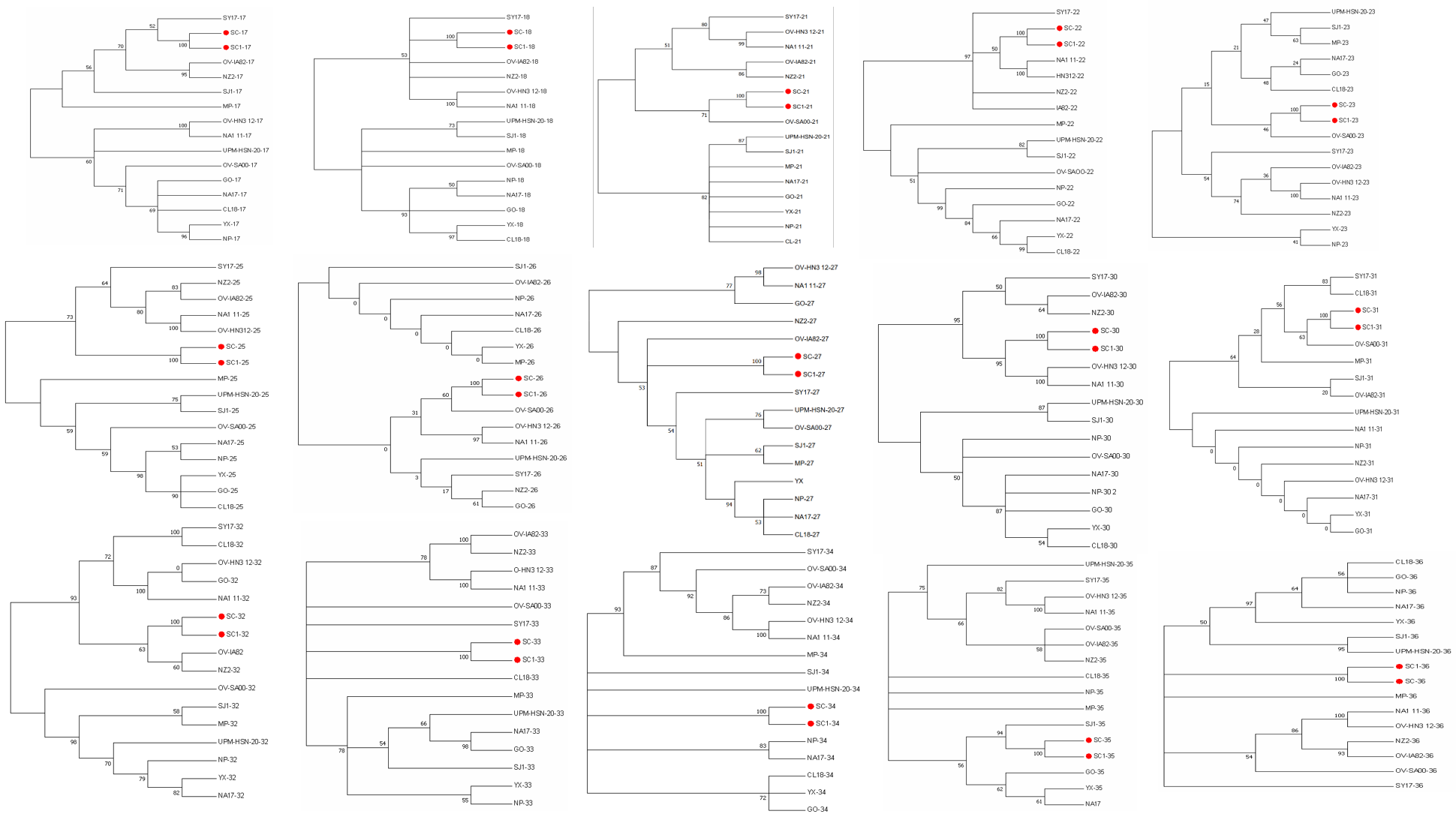

Supplement: Supplementary file 10 — High resolution image (TIF 531 kb) [file 10142_2023_1079_MOESM5_ESM.tif]

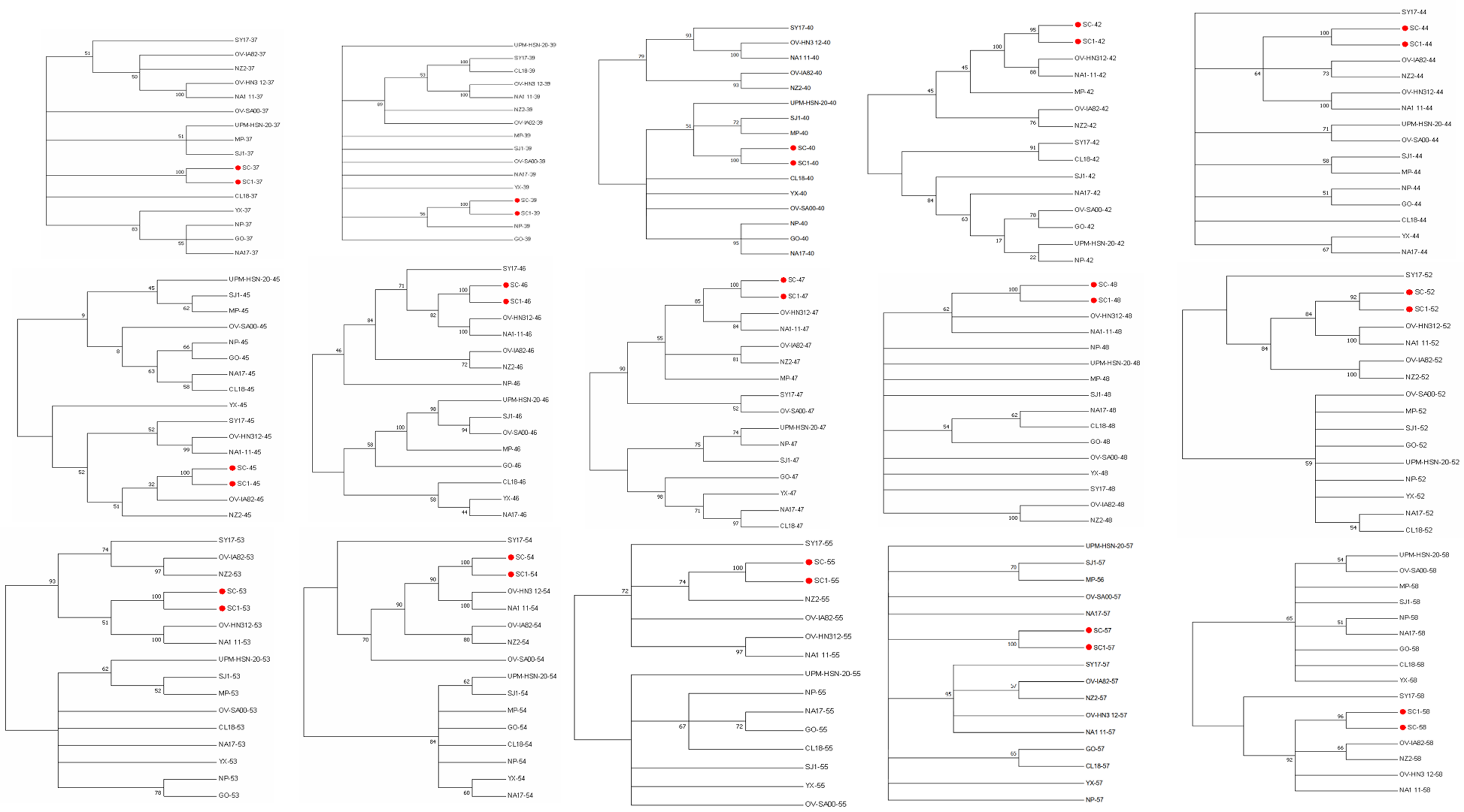

Supplement: Supplementary file 11 — (PNG 763 kb) [file 10142_2023_1079_Fig15_ESM.png]

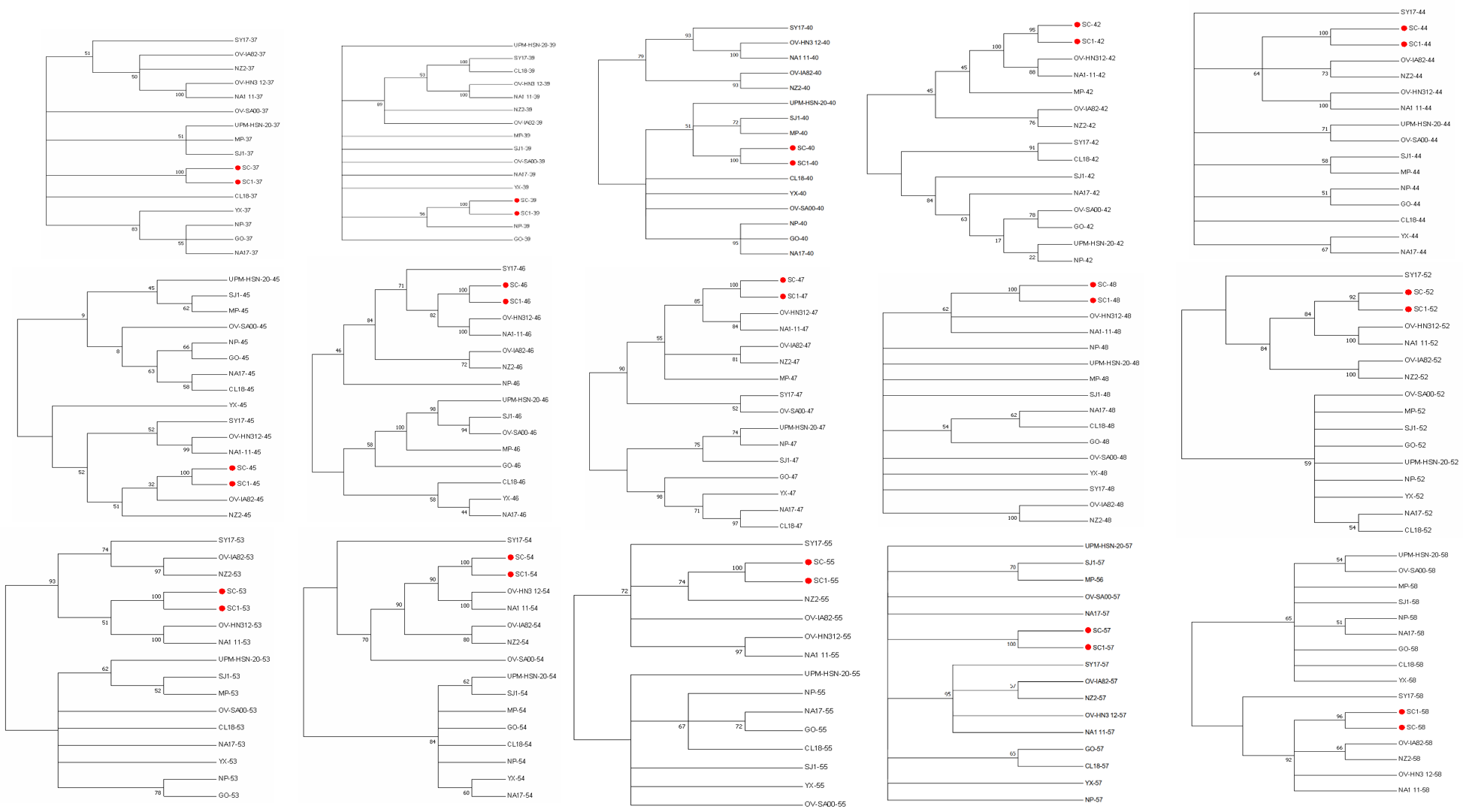

Supplement: Supplementary file 12 — High resolution image (TIF 522 kb) [file 10142_2023_1079_MOESM6_ESM.tif]

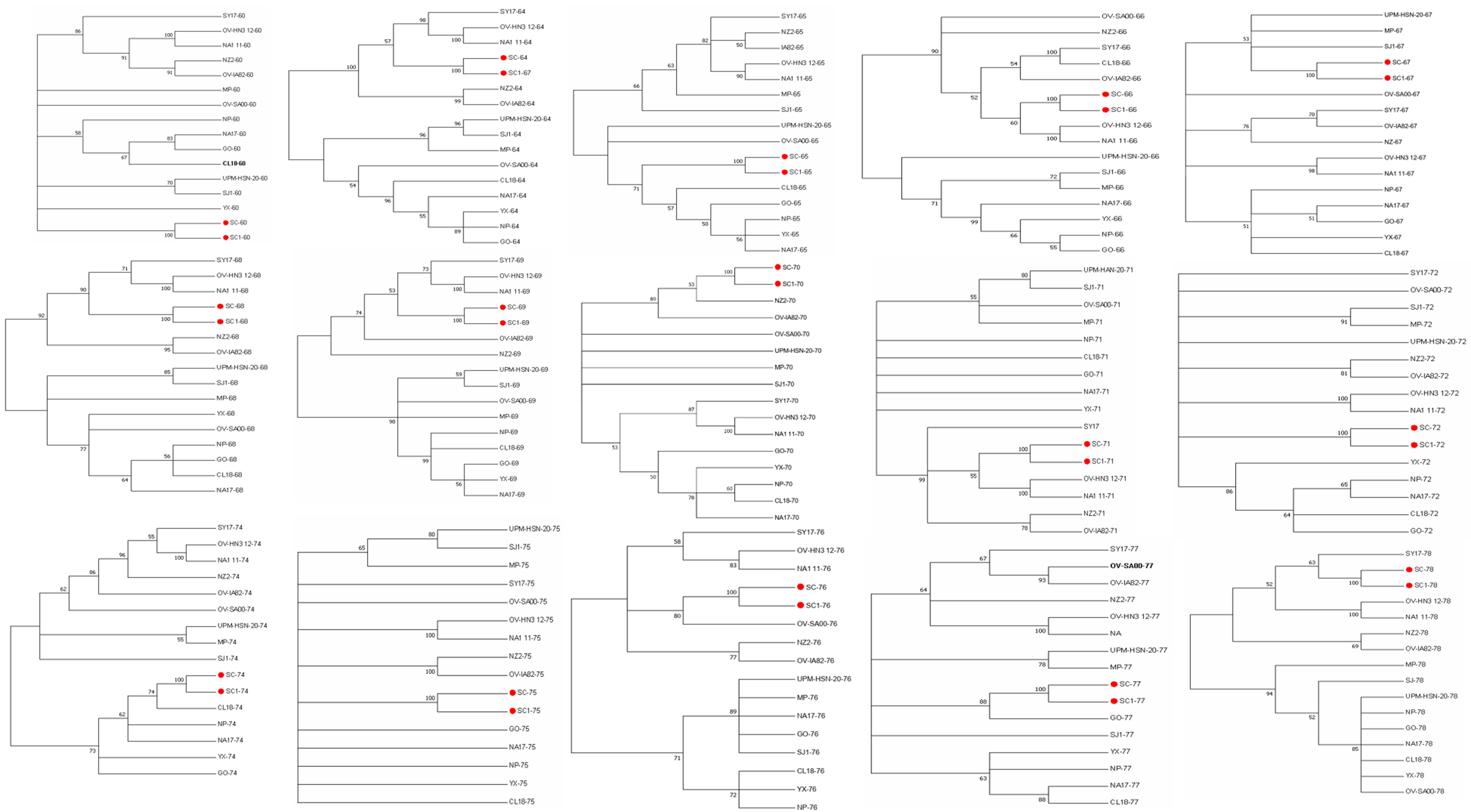

Supplement: Supplementary file 13 — (PNG 810 kb) [file 10142_2023_1079_Fig16_ESM.png]

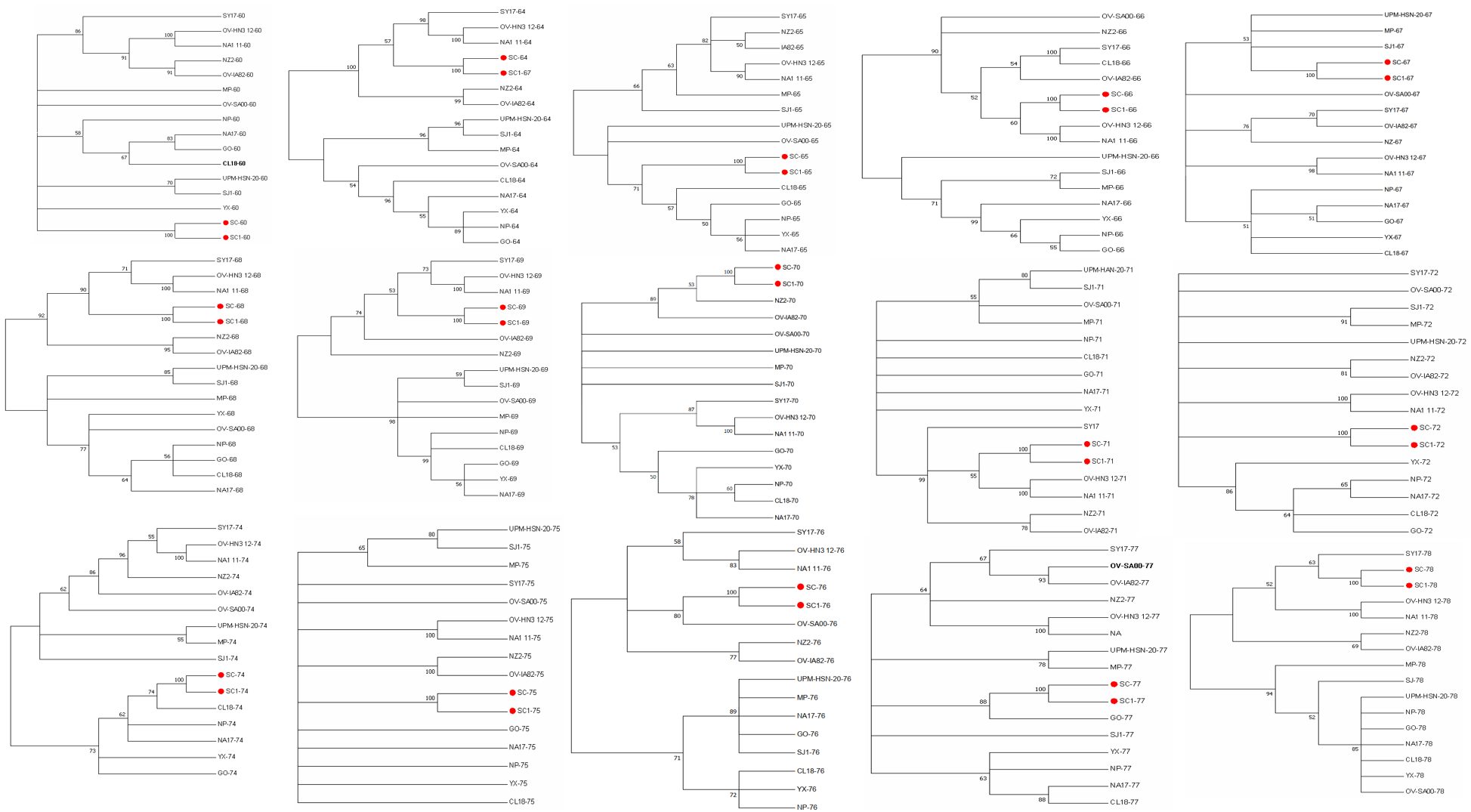

Supplement: Supplementary file 14 — High resolution image (TIF 525 kb) [file 10142_2023_1079_MOESM7_ESM.tif]

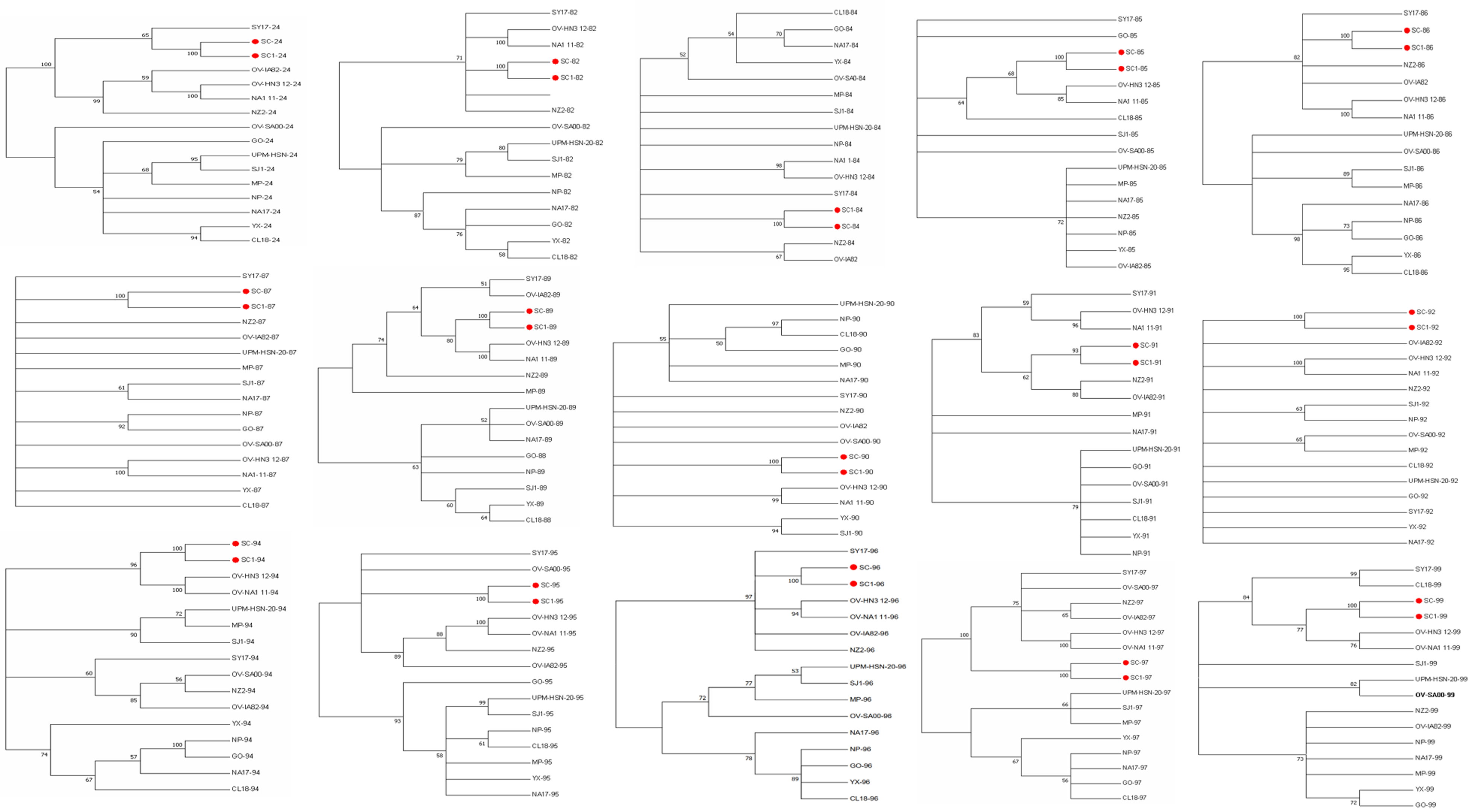

Supplement: Supplementary file 15 — (PNG 764 kb) [file 10142_2023_1079_Fig17_ESM.png]

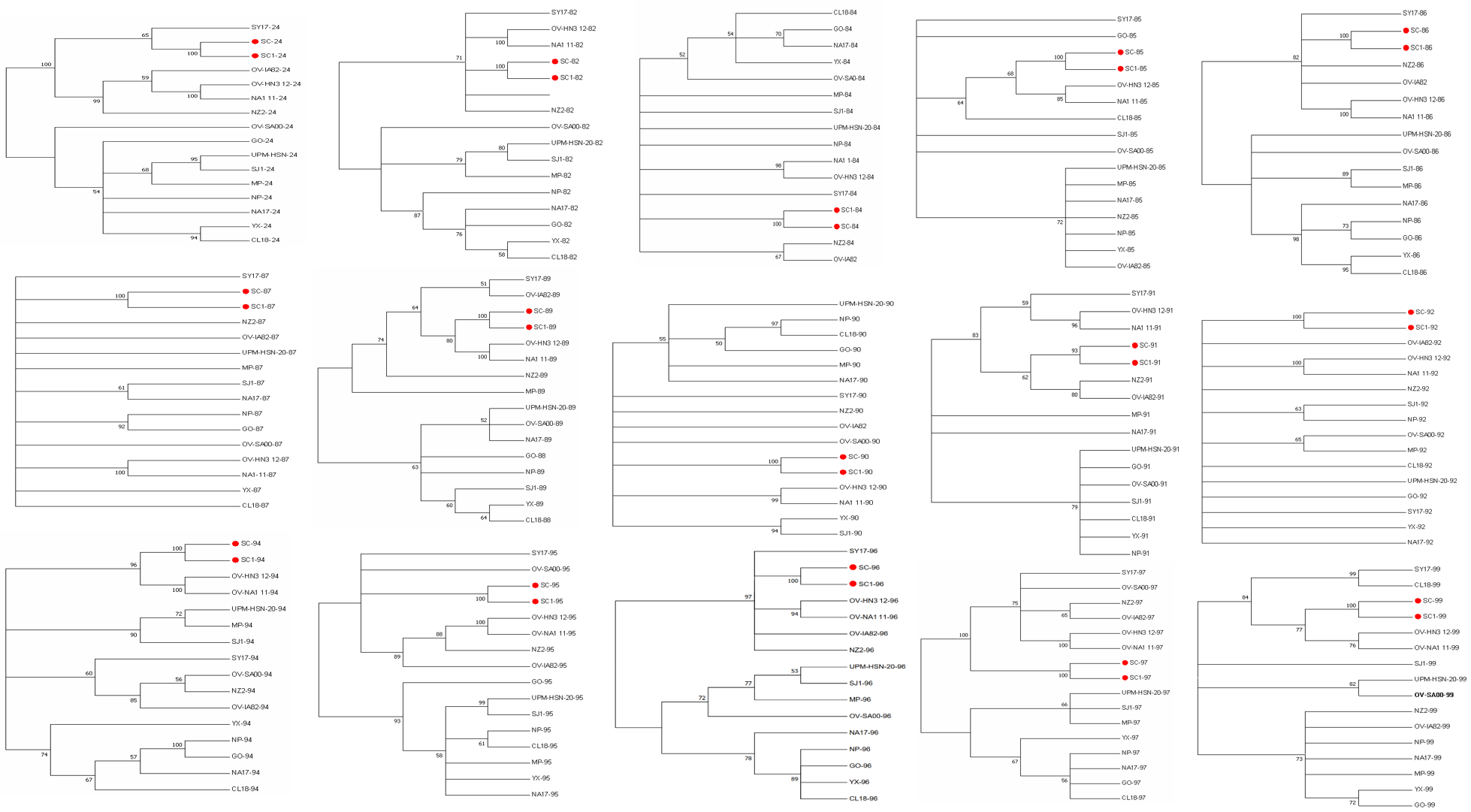

Supplement: Supplementary file 16 — High resolution image (TIF 505 kb) [file 10142_2023_1079_MOESM8_ESM.tif]

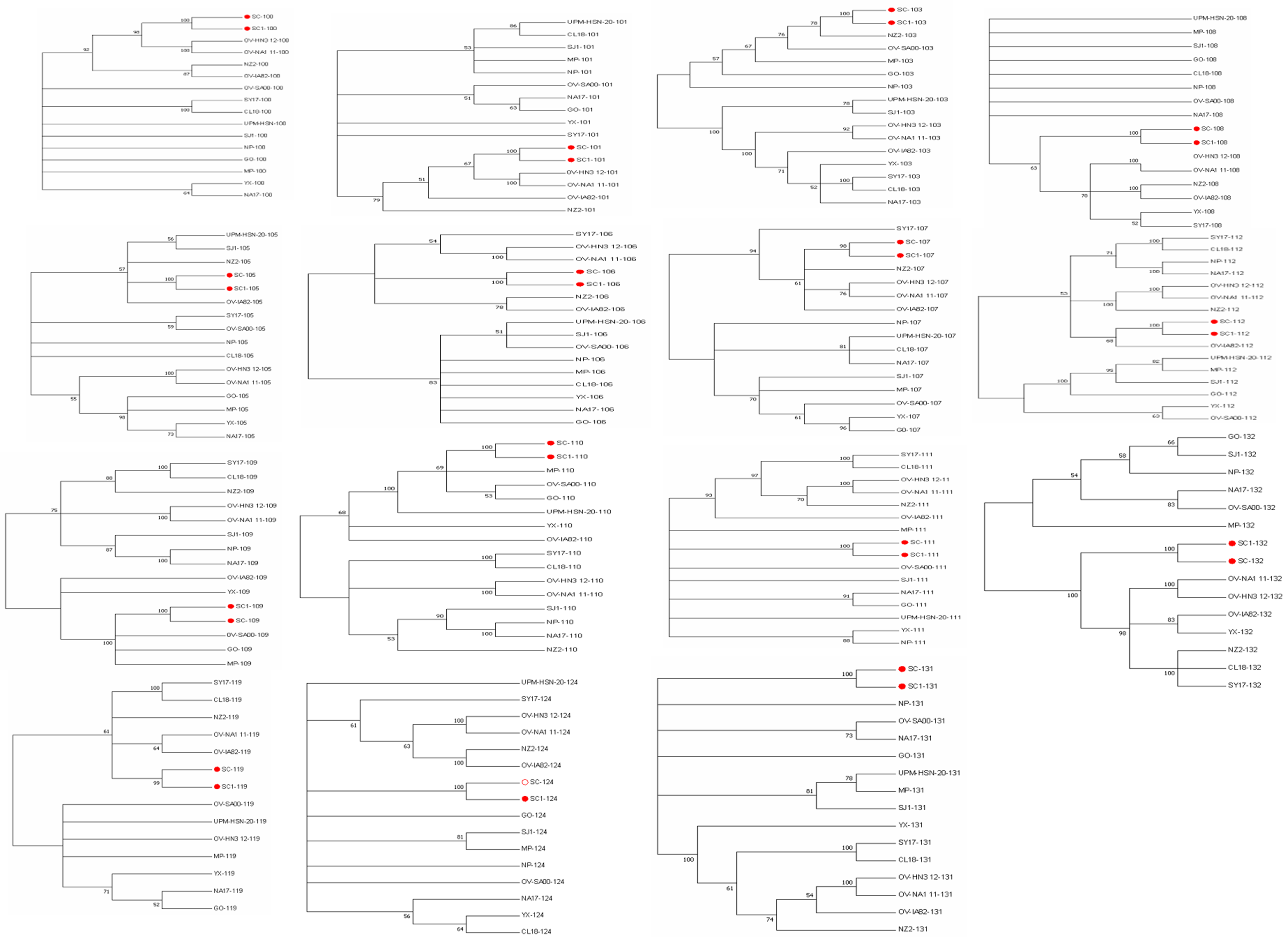

Supplement: Supplementary file 17 — (PNG 856 kb) [file 10142_2023_1079_Fig18_ESM.png]

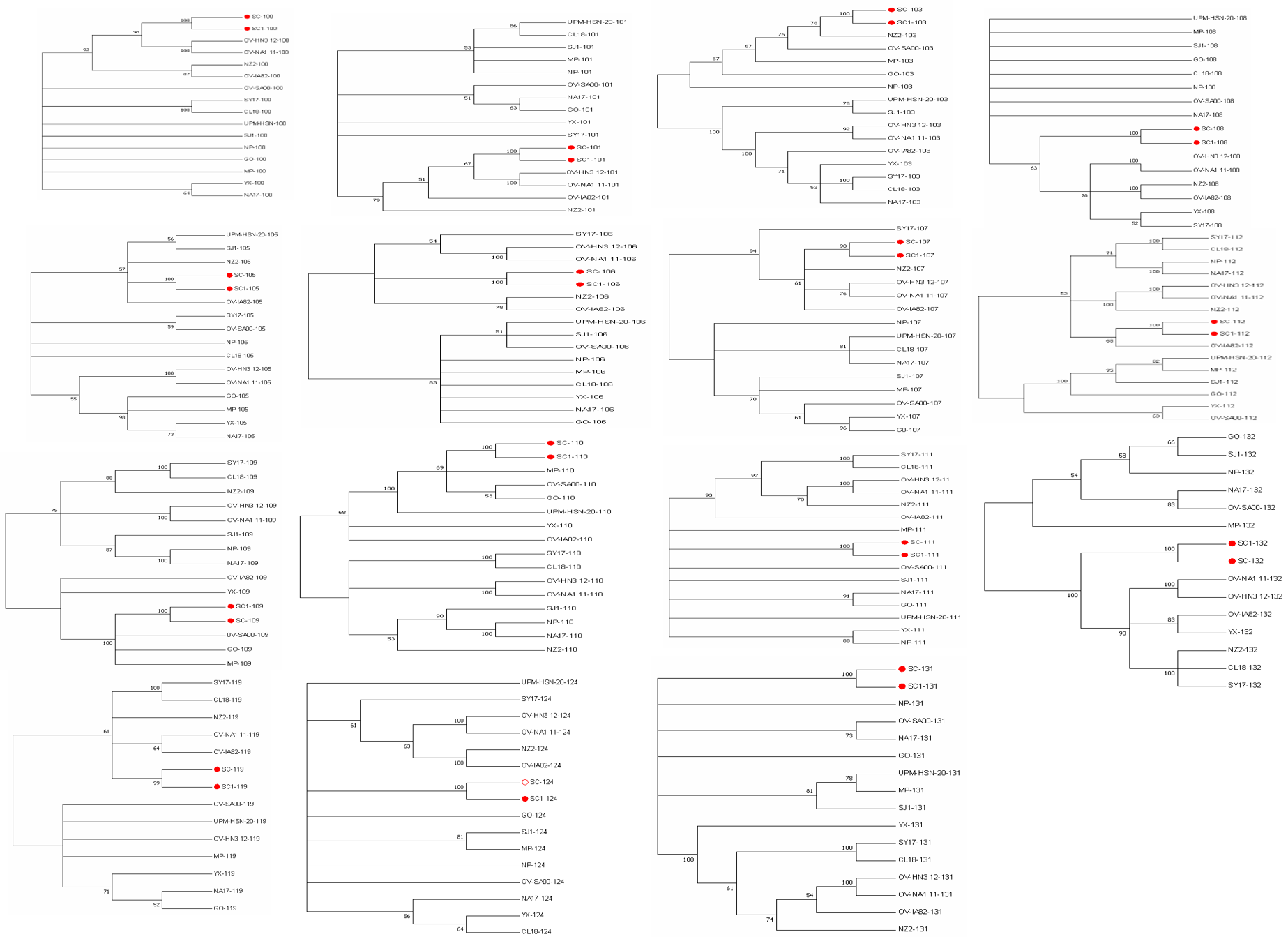

Supplement: Supplementary file 18 — High resolution image (TIF 582 kb) [file 10142_2023_1079_MOESM9_ESM.tif]
