# Supplementary material for: The whole genomic analysis of the Orf virus strains ORFV-SC and ORFV-SC1 from the Sichuan province and their weak pathological response in rabbits
Source: Funct Integr Genomics. 2023 May 16;23(2):163. doi: 10.1007/s10142-023-01079-z (PMC10185592; doi:10.1007/s10142-023-01079-z)
Supplement: Supplementary file 25 — Supplementary Table S4-4 (HTML 16 KB) [file 10142_2023_1079_MOESM16_ESM.html]

NPS@ SOPMA secondary structure prediction results


- PRABI-GERLAND   
  RHONE-ALPES BIOINFORMATIC POLE GERLAND SITE
- Institute of Biology and Protein Chemistry


- Home
- Services
  - Databases search
  - Patterns search
  - Multiple alignment
  - Secondary structure prediction
  - Molecular modeling
  - Software
  - Miscellaneous tools
- Teaching
- Publications
- Links
- Jobs
- Contact

|  |  |
| --- | --- |
|  | Job **SOPMA** (ID: 0de4185151e8) is running on **NPS@** server (started on 20221115-141441). Results will be shown below. **Please wait and don't go back.** |

---

**In your publication cite :**   
NPS@: Network Protein Sequence Analysis  
TIBS 2000 March Vol. 25, No 3 [291]:147-150  
Combet C., Blanchet C., Geourjon C. and Del�age G.  

---

## SOPMA result for : UNK\_111880

Abstract Geourjon, C. & Del�age, G., SOPMA: Significant improvement in protein secondary structure prediction by consensus prediction from multiple alignments., Cabios (1995) 11, 681-684

View SOPMA in:
[AnTheProt (PC)
, Download...]
[HELP]

```
        10        20        30        40        50        60        70
         |         |         |         |         |         |         |
MACECASLILELLRKSDDKLPAKQIAKELGISKLEGNRQLYRLLDSDDVCCEDGNPPRWFVECARSAPIE
hhhhhhhhhhhhhhccccccchhhhhhhhtcchhhhhhhhhhhhtttceeeetccccceeeccccccccc
EEENSDTEPMETEAGCDTLFGGDIDIMTQSSVMRLKSRNLVSAVIEFCMMTHRPLEFCETRSGGKDHCPL
ccccccccchhhhhccccchhccccccchhheehhcccccchhhhhhhhecccceeeeeeccccccccce
FTCTSTISVKVVTAADGASKKLARHTACASAPTILNNNCGISY
eeeeeeetteeeeecttcchhhhhhhhhhhhheeeehttccee
```

```
Sequence length :   183
```

```
SOPMA :
   Alpha helix     (Hh) :    68 is  37.16%
   310  helix       (Gg) :     0 is   0.00%
   Pi helix        (Ii) :     0 is   0.00%
   Beta bridge     (Bb) :     0 is   0.00%
   Extended strand (Ee) :    35 is  19.13%
   Beta turn       (Tt) :    11 is   6.01%
   Bend region     (Ss) :     0 is   0.00%
   Random coil     (Cc) :    69 is  37.70%
   Ambiguous states (?)  :     0 is   0.00%
   Other states         :     0 is   0.00%
```

```
Parameters :
   Window width         :  17
   Similarity threshold :   8
   Number of states     :   4
```

Prediction result file (text): [SOPMA]
  
Intermediate result file (text): [BLASTP on NRPROT] [CLUSTALW]

---

***User :** public@47.57.180.190. **Last modification time :** Tue Nov 15 14:15:06 2022. **Current time :** Tue Nov 15 14:15:06 2022*


- Contact Us
- PBIL, Lyon
- Top of page

Copyright 2016 PBIL-IBCP-Lyon
