# Supplementary material for: The whole genomic analysis of the Orf virus strains ORFV-SC and ORFV-SC1 from the Sichuan province and their weak pathological response in rabbits
Source: Funct Integr Genomics. 2023 May 16;23(2):163. doi: 10.1007/s10142-023-01079-z (PMC10185592; doi:10.1007/s10142-023-01079-z)
Supplement: Supplementary file 28 — Supplementary Table S5-1 (HTML 23 KB) [file 10142_2023_1079_MOESM19_ESM.html]

SMART: Sequence analysis results


- SETUP
- FAQ
- ABOUT
- GLOSSARY
- WHAT'S NEW
- FEEDBACK

Alternative representations: 1 /

Created with Snap PfamPfamdUTPasedUTPase0100

- Architecture

# Domain architecture analysis

## Display all proteins with similar:

- Domain organisation: Proteins having all the domains as the query in the same order. Additional domains are allowed.
- Domain composition: Proteins with the same domain composition have at least one copy of each of domains of the query.

The SMART diagram above represents a summary of the results shown below. Domains with scores less significant than established cutoffs are not shown in the diagram. Features are also not shown when two or more occupy the same piece of sequence; the priority for display is given by **SMART > PFAM > PROSPERO repeats > Signal peptide > Transmembrane > Coiled coil > Unstructured regions > Low complexity**. In either case, features not shown in the above diagram are marked as '**overlap**' in the right side table below.

| Confidently predicted domains, repeats, motifs and features: | Features NOT shown in the diagram: |
| --- | --- |
| | Name | Start | End | E-value | | --- | --- | --- | --- |  |  |  |  |  | | --- | --- | --- | --- | | Pfam:dUTPase | 16 | 130 | 4e-38 |  Click on a row to highlight the feature in the diagram above. Click the feature name for more information. | | Name | Start | End | E-value | Reason | | --- | --- | --- | --- | --- |  |  |  |  |  |  | | --- | --- | --- | --- | --- | | CTNS | 11 | 37 | 1500 | threshold | | PTBI | 42 | 125 | 1150 | threshold |  Click on a row to highlight the feature in the diagram above. Click the feature name for more information. |

� 2021 EMBL | Privacy Policy

Send comments to Ivica Letunic

Selected feature details

MinimizeMaximizeClose
